# An expanded role for the transcription factor *WRINKLED1* in the biosynthesis of triacylglycerols during seed development

**DOI:** 10.3389/fpls.2022.955589

**Published:** 2022-08-05

**Authors:** Cathleen Kuczynski, Sean McCorkle, Jantana Keereetaweep, John Shanklin, Jorg Schwender

**Affiliations:** Biology Department, Brookhaven National Laboratory, Upton, NY, United States

**Keywords:** Arabidopsis, Brassicaceae (= Cruciferae), triacyl glycerol, cis-regulatory element, transcription factor binding motif, seed development, central metabolism, fatty acid biosynthesis

## Abstract

The transcription factor *WRINKLED1* (*WRI1*) is known as a master regulator of fatty acid synthesis in developing oilseeds of *Arabidopsis thaliana* and other species. *WRI1* is known to directly stimulate the expression of many fatty acid biosynthetic enzymes and a few targets in the lower part of the glycolytic pathway. However, it remains unclear to what extent and how the conversion of sugars into fatty acid biosynthetic precursors is controlled by *WRI*1. To shortlist possible gene targets for future *in-planta* experimental validation, here we present a strategy that combines phylogenetic foot printing of cis-regulatory elements with additional layers of evidence. Upstream regions of protein-encoding genes in *A. thaliana* were searched for the previously described DNA-binding consensus for WRI1, the ASML1/WRI1 (AW)-box. For about 900 genes, AW-box sites were found to be conserved across orthologous upstream regions in 11 related species of the crucifer family. For 145 select potential target genes identified this way, affinity of upstream AW-box sequences to WRI1 was assayed by Microscale Thermophoresis. This allowed definition of a refined WRI1 DNA-binding consensus. We find that known WRI1 gene targets are predictable with good confidence when upstream AW-sites are phylogenetically conserved, specifically binding WRI1 in the *in vitro* assay, positioned in proximity to the transcriptional start site, and if the gene is co-expressed with WRI1 during seed development. When targets predicted in this way are mapped to central metabolism, a conserved regulatory blueprint emerges that infers concerted control of contiguous pathway sections in glycolysis and fatty acid biosynthesis by WRI1. Several of the newly predicted targets are in the upper glycolysis pathway and the pentose phosphate pathway. Of these, plastidic isoforms of fructokinase (*FRK*3) and of phosphoglucose isomerase (*PGI*1) are particularly corroborated by previously reported seed phenotypes of respective null mutations.

## Introduction

Triacylglycerol (TAG), the main component of vegetable oils, is an energy dense resource produced by many plants and stored in seeds and other plant organs. Plant oils are important for human nutrition as well as renewable biomaterials and fuels (Chapman and Ohlrogge, 2012). During seed development in oilseed species such as *Arabidopsis thaliana*, TAG is synthesized and accumulated at high rates (Neuhaus and Emes, 2000). Within the developing embryo, sugar supplies (sucrose) provided by maternal tissues are converted by conventional pathways of sugar catabolism into energy cofactors and acetyl-CoA, which is the carbon precursor for chloroplast localized fatty acid synthesis (FAS; Ruuska et al., 2002; O'Grady et al., 2012). In green developing seeds like those of Arabidopsis or *Brassica napus*, photosynthesis can make a significant contribution to oil synthesis *via* re-fixation of CO_2_ and by contribution of energy cofactors derived from the photosynthetic light reactions (Ruuska et al., 2004; Schwender et al., 2004; Goffman et al., 2005; Hay and Schwender, 2011; Borisjuk et al., 2013). *WRINKLED1* (*WRI1*), a transcriptional regulator of the APETALA2/ethylene-responsive element-binding protein (AP2/EREBP) family has been characterized as a seed-specific transcription factor with control over FAS during the synthesis of TAG in developing oilseeds (Focks and Benning, 1998; Cernac and Benning, 2004; Masaki et al., 2005). In addition to seeds, WRI1 has also been shown to control oil biosynthesis in mesocarp tissues of oil palm (Bourgis et al., 2011; Ma et al., 2013). WRINKLED homologs have also been shown to control lipid biosynthesis in a symbiotic context in angiosperms (Jiang et al., 2018; Xue et al., 2018) and in the liverwort *Marchantia paleacea* (Rich et al., 2021). Maeo et al. (2009) identified a consensus for WRI1 binding in upstream regions of WRI1 regulatory target genes, designated ASML1/WRI1 (AW)-box [5’-CNTNG(N)_7_CG-3′, N = A, T, C or G]. Since then, a multitude of studies on lipid biosynthesis in plants rely on the AW-box consensus to assess the presence of WRI1 binding sites in *A. thaliana* and other plant species (recent examples: Adhikari et al., 2016; Guerin et al., 2016; Zhang et al., 2016b; Jiang et al., 2018; Deng et al., 2019; Chen et al., 2020; González-Thuillier et al., 2021; Moreno-Perez et al., 2021; Rich et al., 2021; Sánchez et al., 2022). In a few cases, binding of WRI1 to sites that deviate from the AW-box consensus has been reported (Li et al., 2015; Kim et al., 2016; Kong et al., 2017; Kazaz et al., 2020; Sánchez et al., 2022). Although the AW-box pattern is widely used, 9 of the 14 base positions in the consensus are undefined which means that the current consensus is highly degenerate. Therefore, a reassessment of the WRI1 DNA-binding profile by *in vivo* or *on vitro* methods seems warranted.

*WRI1* is widely understood as a “master regulator” that controls many enzymes involved in the conversion of sugars into fatty acids during development of oilseeds (Baud and Lepiniec, 2008; Chapman and Ohlrogge, 2012; Kong and Ma, 2018). This view is based on several findings. There is good evidence that multiple components involved in the conversion of pyruvate into fatty acids in the plastids are under direct control by WRI1 during seed development (Baud et al., 2007, 2009; Maeo et al., 2009). In addition, it has been well established that WRI1 has direct control over expression of sucrose synthase (Baud et al., 2007; Maeo et al., 2009), which is an entry point into sugar catabolism. WRI1 also strongly induces expression of a subunit of the plastidic pyruvate kinase, which is an end point of glycolysis producing ATP and pyruvate (Andre et al., 2007; Baud et al., 2007, 2009; Maeo et al., 2009). The steps in-between sucrose synthase and pyruvate kinase can be understood as a branched central metabolism network that includes reactions of glycolysis, the oxidative pentose phosphate pathway (OPPP) and the ribulose 1,5-bisphosphate carboxylase/oxygenase shunt (O'Grady et al., 2012). Past studies using metabolic flux analysis and constraint-based modeling gave insights into the coordination of central metabolic pathways in Brassicaceae species to provide biosynthetic precursors and energy cofactors to fuel storage synthesis during seed development (Schwender et al., 2003, 2004, 2006, 2015 Lonien and Schwender, 2009; Hay et al., 2014; Tsogtbaatar et al., 2020). However, for many of the relevant biochemical reactions distinct enzyme isoforms are available, various of which localize to different subcellular locations, and it is not well understood which isoforms might be particularly relevant in the conversion of sucrose to TAG. Given that WRI1 is a global regulator of the conversion of sucrose to TAG, a detailed study of direct gene targets could provide further insight into these issues.

WRI1 and its regulatory control functions appear to be highly conserved across plant species. This is evident from the fact that WRI1 homologues from different species can functionally substitute for each other in inducing oil synthesis in seeds or in leaf tissue (Marchive et al., 2014; Wu et al., 2014; Grimberg et al., 2015; Kong et al., 2019; Yang et al., 2019; Rich et al., 2021). Various findings suggest that WRI1 has specific targets in oil biosynthesis that are conserved across species. For example, co-expression analysis indicates that WRI1 has many of the same (homologous) gene targets during seed oil synthesis in different species (Deng et al., 2019). In several cases where enzymes or proteins with function in lipid metabolism are encoded by multiple isoforms, the same specific isoform tends to be predominantly involved in seed oil biosynthesis in different species (Troncoso-Ponce et al., 2011). If direct regulatory function of WRI1 is conserved across species, functional WRI1 binding sites upstream of lipid biosynthetic genes might also be conserved across species. Identifying such conserved sites would represent an opportunity to identify WRI1 binding sites more reliably. When integrated with additional evidence, such as co-expression information, a more accurate understanding of WRI1 function should emerge.

Here we report the results of a genome-wide search for conservation of the AW-box in 12 species of the mustard family (Brassicaceae), including *A. thaliana* as the reference organism. Among *A. thaliana* genes for which the AW-box was over-represented across orthologous upstream regions (OURs), genes of glycolysis, FAS and TAG biosynthesis were found to be significantly enriched. Furthermore, *A. thaliana* genes for which the AW-box is conserved across species also tend to be co-expressed with WRI1 during seed development. For a sub-set of potential binding sites identified this way, *in vitro* DNA-binding activity to WRI1 protein was determined. Altogether, a metabolic blueprint is presented that gives new insights and additional hypotheses on how *WRI1* orchestrates central metabolism during seed oil biosynthesis.

## Materials and methods

### Data sources and processing

Sequence files corresponding to upstream and downstream sequences of the annotated start codon/end codon of *A. thaliana* were downloaded from The Arabidopsis Information Resource (TAIR; Rhee et al., 2003), version 10 (See details in Supplementary Table 1). For 12 Brassicaceae species used in this study, files for genome sequences, protein sequences and genome annotation (General Feature Format, GFF) were retrieved from sources listed in Supplementary Table 1. In-house generated PERL and PYTHON scripts were used to process and analyze the sequence information. Data processing and analysis was also done using Microsoft Excel^1^ and with Matlab (version R2016a, The MathWorks, Inc., Natick, Massachusetts, United States).

Gene annotation and classification information on *A. thaliana* lipid metabolism genes in was collected from the ARALIP database^2^ (accessed June 29, 2017; Li-Beisson et al., 2013; McGlew et al., 2014). The database contains an expert curated list of 822 reactions/proteins associated with lipid metabolism. 775 AGI gene locus identifiers are associated with 24 lipid pathways.

### Genomic coordinates of transcriptional start sites (TSS)

Information on TSS in the *A. thaliana* genome was obtained by mining published data on 5’-CAP-sequencing (TSS-seq), found in the supplements of Nielsen et al. (2019). Only the wild-type data were used (category “basal”). For peaks residing within the genomic regions “promoter” or “fiveUTR,” the summit position with the highest score associated was taken as TSS. This resulted in TSS positions for 19,505 gene loci. For protein-encoding genes for which this information could not be extracted the start coordinate of the genomic feature “mRNA” was mined from the TAIR10 genome annotation. In a few cases TSS positions were determined based on mRNA sequences deposited in public databases. The position of a motif site relative to the TSS was calculated by taking either the first or the last position of the motif as reference, whichever resulted in the shorter distance.

### Synteny analyses for Brassicaceae genomes and other species

Syntenic orthology relations between protein-encoding genes of *A. thaliana* and other species were derived by using the SynOrths tool (version 1.0, Cheng et al., 2012). In short, this tool derives pairwise synteny relations based on protein sequence similarity and gene adjacencies on contigs (Cheng et al., 2012). For each comparison, the *A. thaliana* genome was always defined as the target genome. Default parameter settings were applied since they have already been optimized for the closely related Brassicaceae genomes (Cheng et al., 2012). In addition to SynOrths outputs, published synteny information related to *B. napus* and *Camelina sativa* was used as additional reference (Chalhoub et al., 2014; Kagale et al., 2014). Within the set of Brassicaceae genomes used here, the genome assembly of *Aethionema arabicum* seemed to be of lesser quality, as judged by the size distribution of contig lengths (Haudry et al., 2013). Therefore, homology relations between the *A. thaliana* genome and *A. arabicum* were derived only based on similarity between sequences of predicted proteins. Protein sequence alignments were established by BLAST (protein sequence similarity; Altschul et al., 1997) with the predicted protein sequences of *A. arabicum* as query against a database of TAIR10 predicted proteins (representative gene models). BlastP results were filtered with an *E*-value cutoff of 10^−7^. Using a custom script, alignments spreading over multiple lines (broken alignments) were joined. Top hits were retained as homology relations but rejected if the alignment length was less than 70% of the length of the query, or if the percentage of identical matches was below 60%.

With regards to possible limitations in tracking synteny relations across genomes, it is notable that the SynOrths approach resolves synteny relations between tandem gene arrays based on arbitrarily selecting one gene as a representant of a tandem arrayed gene family (Cheng et al., 2012). This limitation applies to about 16% of the ortholog sets: For the *A. thaliana* genome, SynOrths identified 4,307 protein-encoding genes (16%) organized in 1638 sets of tandem repeats.

The average composition of sets of ortholog genes is mostly as expected by the ploidy structure of the different genomes (Supplementary Figure 1A). Seven of the 11 relatives of *A. thaliana* in this analysis, like *A. thaliana* itself, are considered diploid and each of those species in average contributes close to one gene to each set of orthologous genes (Supplementary Figure 1B). The polyploid species *Aethionema arabicum*, *B. napus, C. sativa* and *Lepidium meyenii* in average contribute 1.77, 4.17, 2.75, and 3.66 genes, respectively (Supplementary Figure 1B). At least for *C. sativa* and *L. meyenii*, recent polyploids with high gene retention, these contributions are very consistent with their known degree of polyploidy. Relative to the *A. thaliana* genome, there are considered to be three sub-genomes in *C. sativa* (Kagale et al., 2014) and four in *L. meyenii* (Zhang et al., 2016a). The A and C sub-genomes of *B. napus* each are considered to have triplicated genomes relative to *A. thaliana*, but with more substantial loss in gene copies (Chalhoub et al., 2014).

To assess sequence conservation across orthologous upstream regions we used an alignment free pairwise comparison between *A. thaliana* sequences and other species based on *k*mer frequencies (Supplementary Figure 1C). This assessment suggests that dependent on the compared species, the average percent identity ranges between 50% for *Aethionema arabicum* and 70% for *Arabidopsis lyrata* (Supplementary Figure 1D).

Besides the automated analysis, syntenic relationships were tracked manually in a few cases to explore phylogenetic conservation from *A. thaliana* to species outside the Brassicaceae (Supplementary Table 2). Genomic sequences of *Citrus sinensis*, *Daucus carota*, *Populus trichocarpa*, *Ricinus communis*, *Sesamum indicum*, *Sorghum bicolor*, *Tarenaya hassleriana* and *Vitis vinifera* were mined using the online resources of the National Center for Biotechnology Information (NCBI; Coordinators, 2018).^3^ Protein searches among the species was done using NCBI BLAST (Johnson et al., 2008). To identify the gene order in chromosomal neighborhoods of genes of interest of the non-Brassicaceae species, genomic information (GFF files) was accessed at NCBI from sources as listed in Supplementary Table 1.

### Mining of genomic DNA sequences

Nucleotide sequences 500 bp upstream of the ATG start codon were extracted for each protein-encoding gene locus and for each genome of the 12 Brassicaceae species. For this purpose, GFF files (Supplementary Table 1) were mined for genomic coordinates of start codons. The tool gffread (Trapnell et al., 2010) was then used to extract DNA sequences from −1 to −500 nt upstream the start codon from genomic sequences. In case of *A. thaliana*, sequences were extracted only for one gene model per locus (representative gene models). In particular, for each gene in the sequence file “TAIR10_upstream_500_translation_start_20101028” (Supplementary Table 1) start codon positions were identified from sequence headers and matched to one gene model version in the TAIR10 gff file (genomic feature “protein”). This allowed to define genomic coordinates for regions upstream and downstream the transcriptional start codon and downstream the stop codon for each representative gene model.

### Search of DNA sequences for string pattern matches

Searching DNA sequences for all occurrences of a string pattern was done using an in-house tool. The search tool was tested for accuracy by comparing search outputs with the outputs of another pattern search tool^4^ (Stothard, 2000). For each *A. thaliana* gene locus different genomic regions (Upstream and downstream ATG start, intron sequences, downstream stop codon) were searched for occurrence of the AW-box [5’-CNTNG(N)_7_CG-3′, N = A, C, T or G] in both sense and antisense directions. Overlapping motif hits were recognized. For further processing, detected motif matches were recorded along with the gene ID and genomic position of the searched sequence as well as the position and orientation of detected sequences relative to the ATG start.

### *De novo* discovery of conserved motifs

To identify over-represented conserved motifs in genomic upstream regions MEME version 4.11.4 (Bailey and Elkan, 1994) was used with parameters set to: expect zero or one occurrences of the motifs per sequence, request 15 motifs with the width of motifs is 5–25 nucleotides. Background model was 3^nd^ order Markov created using the tool “fasta-get-markov” from the MEME suite (Bailey et al., 2009), using 500 bp upstream regions from 27,416 *A. thaliana* protein-encoding genes.

### Genes associated with the conversion of sucrose to TAG

A metabolic gene set for the conversion of sucrose to TAG was derived considering metabolic pathways as outlined in previous studies (Hay et al., 2014; Schwender et al., 2015) and by using the ARALIP lipid metabolic pathways resource (Li-Beisson et al., 2013). This resulted in a catalog of 309 central metabolism genes associated with sucrose metabolism, glycolysis, the oxidative pentose phosphate pathway, the synthesis, interconversion and degradation of oxaloacetate and malate, biosynthesis and cytosolic elongation of fatty acids as well as triacylglycerol biosynthesis (Columns 1 to 9 in Supplementary Table 3).

### Collection of AW-box sites and assessment of sequence conservation

Nucleotide sequences 500 bp upstream of the ATG start codon from 12 Brassicaceae species were searched for the AW-box binding consensus in both strands and matches were recorded with adjacent sequence context as extended AW-box sites of 18 nc length [5’-NNCNTNG(N)_7_CGNN-3′]. To trace conserved motif instances, AW-box sites found in *A. thaliana* upstream regions were compared to all sites found in OURs (pairwise un-gapped alignments). We considered sequence conservation to be given if two motif instances are identical in orientation relatively to the ATG start and if the sequence comparison of the two 18 nc sequences had at least 15 identities (83.3% identity). This identity threshold was empirically derived by comparison of randomly generated AW-box sites, given a 33.2% G + C content as found in upstream regions of *A. thaliana* (Supplementary Figure 2A). The mean expectation of identities between two random sampled AW-box sites was 8.613 and 15 or more identities were obtained for 0.04% of comparisons, i.e., the by-chance probability to judge two random AW-sites to be conserved is 0.0004 (Supplementary Figure 2A). The distribution of all genome-wide pairwise identities shows that identities of 15 and above occur abundantly but are basically absent if the searched sequences are perturbed by random shuffling (Supplementary Figure 2B). Conserved sequences from multiple pairwise comparisons between an *A. thaliana* AW-box site and orthologous sites were combined into sets of conserved sequences. Per *A. thaliana* gene locus, all conservation relations were aggregated into sets of conserved species (Supplementary Figure 2C). The number of conserved species divided by 12 (total number of assessed species) is the species conservation ratio (Supplementary Figure 2C).

### Sequence logos and computation of PWM scores

Sequence logos were created using the WebLogo version 2.8.2 online tool (Crooks et al., 2004).^5^ The logos created in this study are intended to compare alignments of binding site sequences between each other, not to quantify how degenerate a site is relative to a genomic background. This is important because WebLogo assumes uniform background symbol distribution (all four bases appear with equal background frequency of 0.25), while the genomes studied here have significantly skewed background base distributions. To derive binding affinity scores, PWM scoring matrices were generated (Wasserman and Sandelin, 2004): For each position in a motif nucleotide probabilities were divided by the respective background probabilities derived from *A. thaliana* upstream regions. These values were then converted to log_2_ values. To avoid taking the logarithm of zero, a pseudo-count value of 0.0001 was introduced.

### Enrichment analysis of the presence of AW-box sites in different Arabidopsis genome features

To model the occurrence of a motif in gene regions of uniform size (e.g., 500 bp upstream the ATG start codon), the hypergeometric distribution was applied. One or more matches of the AW-box were counted as a motif hit. If among a total population *N* (total number of searched gene regions) there are *K* motif hits, then the probability to find *m* motif hits in a sub-set of *n* searched gene regions is given by the probability mass function for the hypergeometric distribution:


(1) $$\;P\left(X=m\right)=\frac{\left(\begin{array}{c} K\\ m \end{array}\right)\left(\begin{array}{c} N-K\\ n-m \end{array}\right)}{\left(\begin{array}{c} N\\ n \end{array}\right)}$$

To assess the expected value for *m* AW-motif hits among the *n* = 52 FAS genes, P(*X* = *m*) was computed for *m* ranging within zero and 52 by using the respective Microsoft EXCEL^®^ spreadsheet function (HYPGEOM.DIST). From the results the approximate range of *m* for which 99.9% of the observations are to be expected (99.9% confidence interval) was determined symmetrically around the mean expectation value for *m* (*nK*/*N*).

To test for enrichment of the AW-box across sets of OURs, the cumulative hypergeometric probability mass function was applied. Here *N* is the number of protein-encoding genes for which 500 bp upstream regions were searched among all 12 genomes and *K* is the total number of AW-box hits. In case of overrepresentation (*m* ≥ n*K/*N*), the probability of observing *m* or more AW-box hits within a sample of *n* genes (ortholog group size) is given by:


(2) $$p\left(x\geq m\right)=1-\overset{m-1}{\underset{i=0}{\sum }}\frac{\left(\begin{array}{c} K\\ i \end{array}\right)\left(\begin{array}{c} N-K\\ n-i \end{array}\right)}{\left(\begin{array}{c} N\\ n \end{array}\right)}$$

The false positive rate was estimated with an empirical null model: All *p* value computations were repeated after randomization of the upstream sequences, using the tool “fasta-shuffle-letters” from the MEME suite (Bailey et al., 2009). For a given significance threshold, *t*, the number of significance calls for the randomized sequence data (*p* value ≤ *t*), divided by the number of significance calls for the unperturbed sequence data was taken to be the False Discovery Rate (FDR).

To test for enrichment of the AW-box in *A. thaliana* intron sequences, the sequence file “TAIR10_intron_20101028” was searched. Since the searched DNA sequences were not of uniform size, the hypergeometrical model was not applied. An estimation for the frequency of motif matches by chance was done based on random shuffling of the intron sequences for the 52 FAS genes.

### Pathway and GO-term enrichment analysis

Gene Ontology (GO) term enrichment analysis for sets of Arabidopsis genes (TAIR ID) was performed with the functional annotation tool of the online bioinformatics resources given by the Database for Annotation, Visualization and Integrated Discovery (DAVID; v2021; Jiao et al., 2012).^6^

### Evaluation of publicly accessible gene expression data

A transcriptomic dataset for seed development in *A. thaliana* was taken from literature (Schneider et al., 2016), as public available in the supplemental material of (Hofmann et al., 2019). We used mRNA sequencing data (Illumina reads aligned to TAIR10 genome; transcript per million) of *A. thaliana* wild-type developing embryos at seven time points during seed development, from early bent embryo at 7 days after flowering to post mature green at 17 days after flowering (Schneider et al., 2016). Gene expression values for three experimental replicates were averaged and after adding a pseudo-count of one, averaged values were transformed to the base-2 logarithm. In order to exclude low intensity expressed genes, signals for the lowest 25th percentile of the sum of expression values across the samples were discarded. 20,409 signals remained. Pairwise Pearson’s correlation coefficients were computed between WRI1 (At3g54320) and each of the other signals. Significance (*p* values) of co-expression scores (Pearson’s correlation coefficient, R) was derived based on the distribution of a null model set of R values like in Haberer et al. (2006). First, 1,000 of the 20,409 signals were randomly selected and for each the order of the seven expression values was shuffled. R values were calculated between each of the 1,000 randomized signals and each of the 20,409 unchanged ones. The cumulative distribution function (cdf) of these *R*-values was tabulated. For a given *R*-value, the right tail *p* values are 1 – *cfd*(*R*-value). The *p* values estimated by this method were more conservative than obtained by a modified t-test (Usadel et al., 2009).

### Co-expression network analysis

To retrieve WRI1 (AT3G54320) co-expressed genes the gene co-expression database ATTED-II (Version 11.0) was accessed (Obayashi et al., 2007, 2022).^7^ The co-expression measures obtained from the database are z-scores, which are derived from *A. thaliana* gene expression dataset “ath-u.2.” The dataset unifies microarray and RNA-seq gene expression data (Ath-m.c9-0, Ath-r.c5-0), covering 862 experiments with 27,427 samples for 18,957 genes. For a given query gene, the database returns a ranked list of co-expressed genes. A co-expression network was derived as follows. With WRI1 as the primary gene of interest, we first defined the five top-ranking WRI1 co-expressed genes as guide genes. Each of the guide genes was then linked to the 50 highest-ranking genes taken from its respective co-expression gene lists. We then derived a co-expression network module by removing all newly added nodes that are connected by only one edge, which removed 155 of the 250 newly added edges. This resulted in a WRI1 associated co-expression module with 47 nodes (genes) and 132 edges (Supplementary Figure 3).

### Microscale thermophoresis

Specific binding of AW-sites by AtWRI1 was measured by microscale thermophoresis (MST; Seidel et al., 2013). A genetic construct combining the coding region responsible for DNA binding, green fluorescent protein (GFP) and a HIS-tag (*At*WRI_150-240_-GFP-HIS) was expressed in *E. coli* and the protein purified as described before (Liu et al., 2019). Thermophoretic assays were conducted using a Monolith NT.115 apparatus (NanoTemperTechnologies, South San Francisco, CA).^8^ Assay conditions were as previously (Liu et al., 2019). dsDNA oligomers were hybridized using a thermal cycler. For determining an equilibrium dissociation constant (*k_D_*), 16 reactions were prepared: 8 nM of-AtWRI1_58-240_-GFP was incubated with a serial (1:1) dilution of the ligand (dsDNA) from 1.25 μM to 38.81 pM or from 6.25 μM to 190 pM. Samples of approximately 10 μl were loaded into capillaries and inserted into the MST NT.115 instrument loading tray. All thermophoresis experiments were carried out at 25°C using 40% MST power and 100% or 80% LED power. The data were fitted with the NanoTemper Analysis software v2.2.4. To ensure reproducibility, any series of measurements performed at 1 day included a reference DNA probe (Ligand 10, Supplementary Table 4). For this ligand the average binding affinity (*k_D_*) out of multiple series of measurements was 7.0 ± 3.5 nM. In each series of measurements, *k_D_* values given by the analysis software were only accepted if the response amplitude was within about ±20% of the response amplitude measured for the reference DNA probe (Supplementary Figure 4). Otherwise, the DNA fragment was assessed to be “not binding.” For examples of evaluation of analysis MST results see Supplementary Figure 4. Statistics for *k_D_* values (standard deviation) were derived from three measurements of a DNA ligand obtained from different measurement series.

## Results

### The AW-box is enriched in −1 to 500 bp upstream regions among fatty acid biosynthetic genes in *Arabidopsis thaliana*

The AW-box consensus was first identified by comparing 7 WRI1 binding promoter fragments upstream of fatty acid biosynthetic genes in *A. thaliana* (Maeo et al., 2009). Searching the consensus in 1000-bp upstream regions of other FAS genes, Maeo et al. (2009) located 26 AW-box sites in of 19 out of 46 genes they identified as FAS genes, suggesting that the AW-box is enriched specifically in the upstream regions of FAS pathway genes. We therefore set out to test for enrichment of the motif upstream ATG and in upstream regions and other genomic features for 52 *Arabidopsis* genes annotated by the ARALIP database to be involved in FAS (annotation ‘Fatty Acid Synthesis’; Li-Beisson et al., 2013), relative to the genomic background (Table 1). AW-box pattern matches were collected within six different search windows, defined relative to the position of the start codon as well as the stop codon of the gene models (Table 1). Any number of pattern matches per searched sequence was counted as a hit. 30 hits were found for the −1 to −500 bp upstream region of the 52 FAS genes, which is significantly above the genomic expectation of 10.5 (hypergeometric *p* value 3 × 10^−9^; Table 1). The AW-box was not over-represented in any of the other searched regions (Table 1). This also applies to intron sequences, which were tested slightly differently because of their variable length (Supplementary Table 5B). Since for the −1 to −500 bp upstream region the mean expectation (10.5) amounts to 35% of the detected number of the hits upstream of FAS genes (30), a substantial fraction of the 30 observed hits could be false positive. In addition, the number of genome-wide motif hits in the 500 bp upstream region (5540) is very similar to the number of hits in controls with random sequences (Table 1), suggesting that most motif hits across the genome are spurious matches and likely not functional. Together, the statistical evaluation in Table 1 shows that the AW-box is enriched in the −1 to −500 bp upstream regions of fatty acid biosynthetic genes. This indicates that target sites of WRI1 are concentrated in this genomic region. Therefore, in the following, the cross-species search for AW-box sites focused on the upstream region from −1 to −500 bp.

**Table 1:** Enrichment of the AW-box in genomic features of genes encoding for fatty acid biosynthesis in *Arabidopsis thaliana*.

Genomic feature relative to start/stop codons	Genome-wide number of hits (frequency of hits [%])	Mean expected number of hits for 52 draws [99.9% confidence range]	Number of hits for 52 FAS genes	Fold enrichment	Hypergeometric *p* value
1 to 500 bp upstream start	5,540 (20.2)^1^	10.5 [0, 20]	30	2.85	3 × 10^−9^
501 to 1,000 bp upstream start	5,675 (20.7)^1^	10.8 [1, 21]	12	1.11	0.39
1,001 to 1,500 bp upstream start	6,188 (22.6)^1^	11.7 [1, 21]	12	1.02	0.52
500 bp downstream start	11,414 (41.6)^2^	21.7 [10, 32]	25	1.15	0.21
500 bp downstream stop	4,569 (16.7)^2^	8.7 [0, 18]	6	0.69	0.88
Randomized controls for regions upstream start codon					
1 to 500 bp upstream start^3^	5,919 (21.6)	11.2 [1, 21]	9	0.80	0.82
random sequences^4^	5,889 (21.5)	11.2 [1, 21]	n/a	n/a	n/a

For all protein-encoding genes, regions relative to the start or stop codon were searched for the AW-box pattern. The presence of the AW-box (AW-box hits) within the set of 52 FAS genes was modeled by the hypergeometric distribution based on the genome-wide background. More details and controls see Supplementary Table 5.

1 Average GC content close to 33%.

2 The frequency of hits is different from the upstream regions, which is explainable by differing GC contents in these regions. See additional controls for variability on GC content in Supplementary Table 5A.

3 Sequences were randomly shuffled using the “fasta-shuffle-letters” tool from the MEME suite (Bailey et al., 2009) with default settings.

4 Pseudo-random 500 bp sequences generated with the FaBox online tool (http://users-birc.au.dk/palle/php/fabox/random_sequence_generator.php; Villesen, 2007) were generated using the average GC content in the −1 to −500 bp upstream sequences as input (33%).

### Synteny analysis across 12 *Brassicaceae* genomes

To allow testing for enrichment and conservation of DNA sequence motifs across orthologous promoter regions, genomic information for 12 species within the Brassicaceae family was collected from public available sources, including *A. thaliana*, which was designated as the reference organism (Table 2). To derive synteny relations, we compared the *A. thaliana* genome to each of the other genomes in a pairwise fashion using the SynOrths tool (Cheng et al., 2012). The process defines synteny based on the chromosomal order of genes in the compared genomes and on sequence similarity of the encoded polypeptides (see methods). In result, for all species between 70 and 94% of the genes were found in syntenic orthology relation (Table 2), which is similar to the 68 to 92% of *A. thaliana* protein-encoding gene orthologs previously reported for *Brassicaceae* genomes (Haudry et al., 2013) and confirms that *Brassicaceae* genomes tend to be highly syntenic. All pairwise orthology relations were further aggregated into 25,545 sets of orthologous genes. Each set is identified by the *A. thaliana* gene ID within the set. The median size of ortholog gene sets is 18 (Supplementary Figure 1A), which is higher than the number of contributing species. The discrepancy is explained by the fact that three of 12 species are polyploid and contribute, on average, more than one gene to each orthologous gene set (see Methods).

**Table 2:** Species used in the phylogenetic foot printing approach and main results of genome mining for syntenic orthology relations and promoter regions.

Species	Subfamily/tribe^1^	Gene count^2^	Percentage of genes in orthology alignment	Genes with AW-motif hit 500 bp upstream ATG start	Frequency of hits [%]	Genome publication
*Arabidopsis thaliana*	Camelineae^3^	27,414	93	5,540	20.2	Lamesch et al., 2012
*Arabidopsis lyrata*	Camelineae	31,065	73	6,534	21.0	Rawat et al., 2015
*Camelina sativa*	Camelineae	89,285	70	17,428	19.5	Kagale et al., 2014
*Capsella grandiflora*	Camelineae	24,774	81	4,673	18.9	Slotte et al., 2013
*Capsella rubella*	Camelineae	26,519	79	5,400	20.4	Slotte et al., 2013
*Cardamine hirsuta*	Cardamineae^4^	29,452	75	6,206	21.1	Gan et al., 2016
*Lepidium meyenii*	Lepidieae^4^	96,413	72	26,733	27.7	Zhang et al., 2016a
*Brassica napus*	Brassiceae^4^	101,039	94	23,217	23.0	Chalhoub et al., 2014
*Thellungiella halophila (Eutrema halophilum)*	Eutremeae^4^	26,349	75	5,769	21.9	Yang et al., 2013
*Thellungiella parvula (Schrenkiella parvula)*	Eutremeae	25,655	75	5,614	21.9	Dassanayake et al., 2011
*Thlaspi arvense*	Thlaspideae^4^	27,389	70	6,318	23.1	Dorn et al., 2013
*Aethionema arabicum*	Aethionemeae^5^	37,722	77	7,560	20.0	Haudry et al., 2013

More details on data sources see Supplementary Table 1.

1 Taxonomic classification according to Franzke et al. (2011).

2 Count of protein-encoding gene IDs for which 500 bp upstream regions were extracted.

3 Lineage I of Brassicaceae.

4 expanded lineage II of Brassicaceae.

5 lineage basal to the major three Brassicaceae lineages.

### AW-box signatures are significantly enriched and conserved across orthologous upstream regions of *Arabidopsis thaliana* genes and particularly for many genes of fatty acid biosynthesis and glycolysis

**Figure 1:**
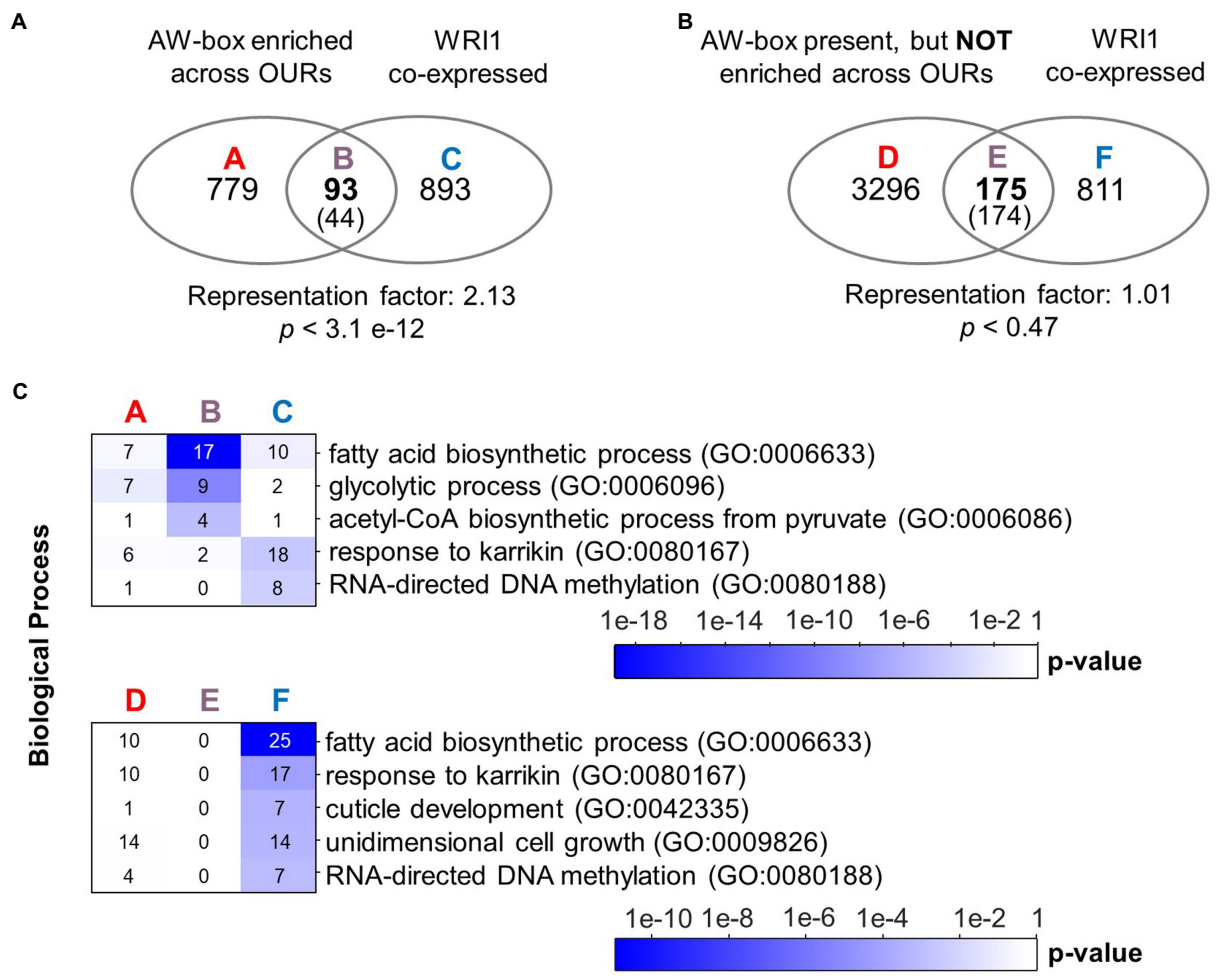
Overrepresentation of the AW-box across orthologous upstream regions identifies likely WRI1 gene targets. **(A)** Overlap of *A. thaliana* genes with AW-box enriched across OURs and genes that are found to be co-expressed. In the intersect, expectation values are shown in parentheses. Statistical significance is indicated by hypergeometric *p* value. **(B)** Venn diagram like panel A, but considering only genes for which the AW-box is present upstream *A. thaliana* genes but not enriched across OURs. **(C)** Overrepresentation of GO biological processes among sets A to E is shown in panels A and B. Numbers on the heat maps are gene counts. A seed development transcriptomic dataset of Schneider et al. (2016) was mined (see methods). Co-expressed genes are defined by a cutoff for the Pearson correlation coefficient (*R*-value) of 0.943. Similar results are obtained for a range of threshold values tabulated in Supplementary Table 11. GO term analysis was performed using the DAVID version 6.8 online resource (https://david.ncifcrf.gov/; Huang da et al., 2009). Abbreviation: OUR, orthologous upstream region.

To search for conserved motifs, 543,076 genomic regions between −1 and − 500 bp upstream the translational start of protein-encoding genes were extracted across the 12 *Brassicaceae* species of this study. Next, the AW-box pattern was searched for all extracted genomic regions (Table 2). The AW-box was found for in-between 19.5 and 23% of the upstream sequences (Table 2), which is similar to the frequency of 20.2% in *A. thaliana* (Table 1). Overrepresentation of AW-box hits among orthologous upstream regions (OURs) was tested for based on the cumulative hypergeometric probability function (Methods). The False Discovery Rate (FDR) was estimated based on re-determination of the hypergeometric *p* values if the pattern search was repeated with random shuffled upstream sequences (Supplementary Figure 5A). For a *p* value threshold of 3.02 × 10^−4^ the empirical FDR was 5%. In this case, the AW-box was judged to be significantly enriched across OURs for 915 *A. thaliana* genes (Supplementary Table 6), which is 16.5% of the 5,540 genes for which the AW-box was detected (Table 1). GO term analysis of the enrichment gene set showed significant overrepresentation of genes involved in fatty acid biosynthesis (p value 4.1 × 10^−10^) and glycolysis (p value 1.7 × 10^−8^; Table 3). In addition, considering a transcriptomic dataset of *A. thaliana* developing embryos (Schneider et al., 2016; see methods), the AW-box enrichment gene set was found to have significant overlap with genes that are co-expressed to WRI1 during seed development (2.1-fold enrichment, *p* value 3.1 × 10^−12^; Figure 1A). Functions associated with fatty acid biosynthesis and glycolysis are enriched particularly in the intersect between the AW-box enrichment gene set and WRI1 co-expressed genes (Figure 1C). If the overlap with WRI1 co-expressed genes is made with *A. thaliana* genes for which the AW-box is present but not significantly enriched across OURs, the number of genes in the overlap is close to the expectation for randomized gene sets (Figure 1B) and in this case the intersect in the Venn diagram is not enriched with genes of fatty acid synthesis or glycolysis (Figure 1C). These findings strongly suggest that WRI1 gene targets are frequently associated with conserved AW-box sites, whereas the presence of an AW-box site alone often does not reliably identify gene targets. To assure that these findings depend on the AW-box pattern, we repeated the gene set enrichment and overlap analysis of Figures 1A,B for 10 modifications of the AW-box search pattern (random base substitutions and permutations; Supplementary Figure 5B). For the modified search pattern, the size of the gene overlaps was always close to the random expectation (Supplementary Figure 5C). Altogether, the overlap with WRI1 co-expressed genes in Figure 1 indicates that overrepresentation of the AW-box in OURs identifies likely WRI1 gene targets, among which genes of fatty acid biosynthesis and glycolysis are particularly overrepresented.

**Table 3:** GO term enrichment for 915 *A. thaliana* genes for which the AW-box is significantly over-represented in in the 500 bp upstream region and orthologous upstream regions (OURs).

Biological Process	Gene count	Fold Enrichment	*p* value^*^
Fatty acid biosynthetic process (GO:0006633)	24	4.97	4.08E-10
Glycolytic process (GO:0006096)	16	6.42	1.67E-08
Acetyl-CoA biosynthetic process from pyruvate (GO:0006086)	5	14.27	2.45E-04
Gluconeogenesis (GO:0006094)	7	7.20	3.19E-04
Seed maturation (GO:0010431)	6	5.01	9.29E-04

GO term analysis was performed using the DAVID version 2021 online resource (https://david.ncifcrf.gov/; Huang da et al., 2009). *A. thaliana* genes that identify 25,545 sets of orthologous genes were set as background. Shown are the five top ranking terms for Biological Process. See also Supplementary Table 6.

* Adjusted *p* value (Bonferroni correction).

**Figure 2:**
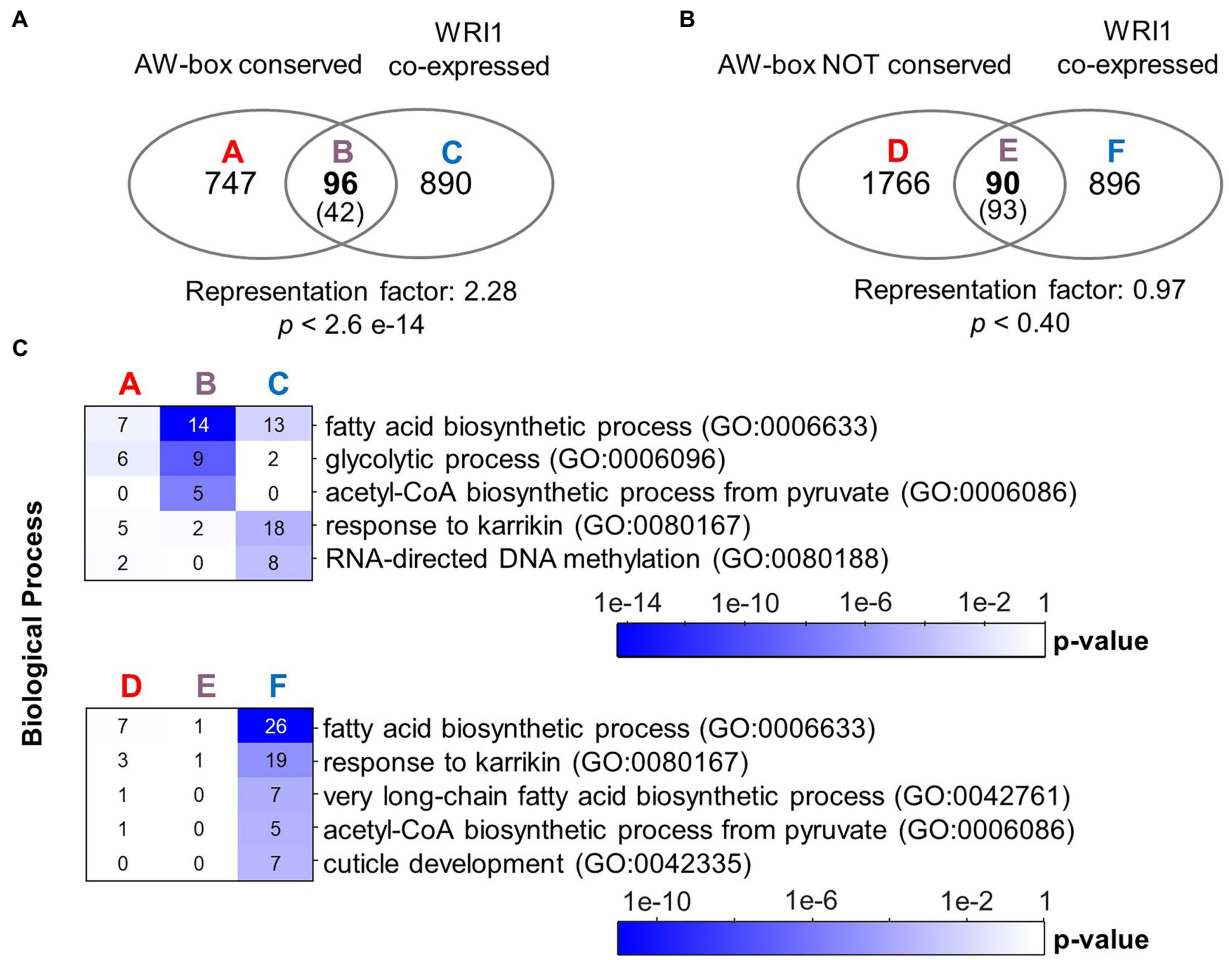
Conservation of the AW-box across orthologous upstream regions identifies likely WRI1 gene targets. **(A)** Overlap of *A. thaliana* genes with AW-box conserved in five or more other species (Species conservation ratio ≥ 0.5). In the intersect, expectation values are shown in parentheses. Statistical significance is indicated by hypergeometric *p* value. **(B)** Venn diagram like panel A, but considering only genes for which the AW-box is present upstream *A. thaliana,* but no AW site is conserved (Species conservation ratio 0). **(C)** Overrepresentation of GO biological processes among sets A to E is shown in panels A and B. Numbers on the heat maps are gene counts. A seed development transcriptomic dataset of Schneider et al. (2016) was mined (see methods). Co-expressed genes are defined by a cutoff for the Pearson correlation coefficient (*R*-value) of 0.943. GO term analysis was performed using the DAVID version 6.8 online resource (https://david.ncifcrf.gov/; Huang da et al., 2009). Abbreviation: OUR, orthologous upstream region.

The appeal of the above analysis of motif enrichment in sets of orthologous sequences is that the FDR can be determined with a simple randomized model. However, detection of the presence/absence of motif hits used in the analysis does not reveal to which degree AW-box sites are conserved. Due to the degeneracy of the AW-box (5 invariable base positions, 9 “N” positions), two motif matches found in OURs may have as few as 5 identical bases along the 14 nc binding motif site (36% identity) or might be located on opposite DNA strands. Therefore, as an alternative approach, an analysis of the degree of sequence conservation of AW-box sites was undertaken based on a pairwise sequence comparison between AW pattern matches detected in upstream regions of *A. thaliana* and those in orthologous sequences. Assuming that sequence conservation between motif sites should extend somewhat into the flanking regions of the motif pattern on either side, all sequence comparisons were performed for AW-box sites that were extended by two adjacent bases on each side. We defined that there is a conservation relationship between two extended AW-sites if at least 15 out of 18 bases are identical (83% identity) and if the two compared motif matches occur in identical DNA strand orientation relative to the direction of transcription (see Methods). Per *A. thaliana* gene locus, conservation relations between individual AW-sites were aggregated onto the species level so that AW-sites can be found to be conserved between two and all 12 species of this study. We define a species conservation ratio as the number of species in conservation relationship divided by 12, the number of species in this study (see Methods). For a species conservation ratio of 0.5 and above, 889 potential target genes are identified (listed in Supplementary Table 7), which is close to the number of 915 genes obtained in the motif overrepresentation analysis. GO term analysis for this conservation gene set again showed significant overrepresentation of genes involved in fatty acid biosynthesis (*p* value 9.6 × 10^−06^) and glycolysis (p value 9.1 × 10^−5^; Table 4). The conservation gene set is not identical to the above AW-box enrichment gene set but has substantial overlap with it (601 genes intersect, 18.9-fold above expectation, p value <1 × 10^−300^). Also, in similar to the overlap analysis as shown in Figure 1A, there is an overlap above expectation between the conservation gene set and WRI1 co-expressed genes (2.3-fold enrichment, *p* value 2.6 × 10^−14^, Figure 2A). On the other hand, there is no significant overlap between genes for which the AW-box is present but not conserved and WRI1 co-expressed genes (enrichment factor 0.97, value of *p* is 0.40; Figure 2B). Functions associated with fatty acid biosynthesis and glycolysis are enriched particularly in the intersect between WRI1 co-expressed genes and genes with conserved AW-box, but not when the intersect is made with genes for which the AW-box is not conserved (Figure 2C).

**Table 4:** GO term enrichment for 889 *A. thaliana* genes for which the AW-box is present in the 500 bp upstream region and conserved in at least 5 out of the 11 other species (OURs).

Biological Process	Gene count	Fold Enrichment	*p* value^*^
Fatty acid biosynthetic process (GO:0006633)	21	4.94	9.64E-06
Glycolytic process (GO:0006096)	15	6.17	9.09E-05
Acetyl-CoA biosynthetic process from pyruvate (GO:0006086)	5	14.64	1.98E-01
Abscisic acid-activated signaling pathway (GO:0009738)	20	2.49	3.73E-01
Seed maturation (GO:0010431)	7	6.59	4.14E-01

GO term analysis was performed using the DAVID version 2021 online resource (https://david.ncifcrf.gov/; Huang da et al., 2009). *A. thaliana* genes that identify 25,545 sets of orthologous genes were set as background. Shown are the five top ranking terms for Biological Process. See also Supplementary Table 7.

* Adjusted *p* value (Bonferroni correction).

### *De novo* prediction of possible WRI1 binding motifs discovers an AW-box like motif

**Figure 3:**
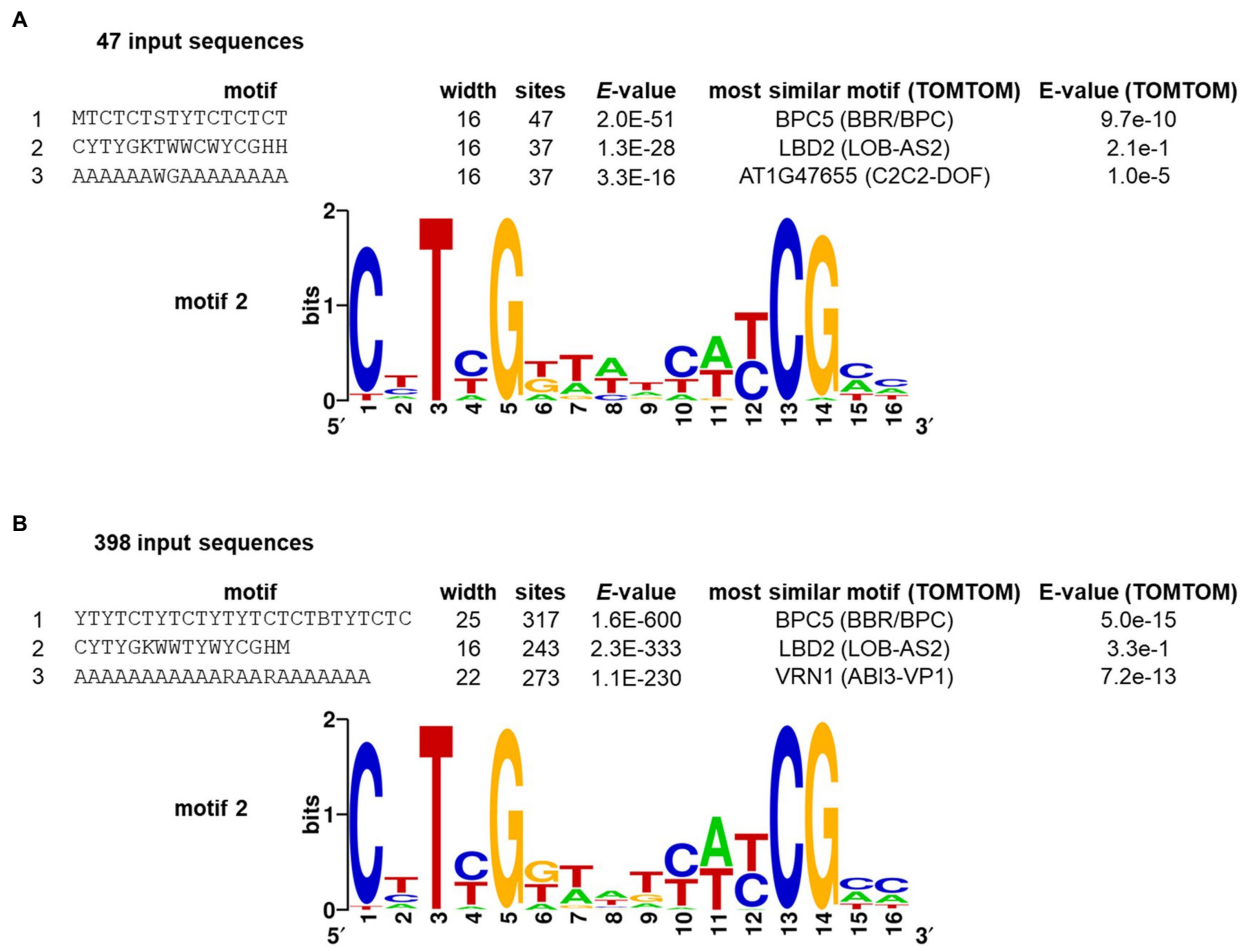
*De novo* discovery of cis-elements upstream of WRI1 co-regulated genes by the MEME algorithm, requesting 15 motifs of length 5 to 25 nc. WRI1 targets were identified by a co-expression network (Supplementary Figure 3). Listed motifs from the MEME output were searched against *A. thaliana* DAP-seq motifs (O'Malley et al., 2016) using the motif comparison tool TOMTOM of the MEME suite (Bailey et al., 2009). **(A)** motifs detected in upstream regions of 47 *A. thaliana* genes found to be co-expressed to WRI1 (see text). Motifs with *E*-value <1E-10 are listed. **(B)** motifs detected in a set of 398 upstream regions. The 47 sequences from panel A were extended by 351 orthologous upstream regions from other species. The three top-ranking motifs from the MEME output are listed.

Our analysis of sequence enrichment and conservation described here relies on a previously described search pattern. Since the AW-Box consensus was originally derived as agreement of only seven experimental confirmed binding sites (Figure 7f in Maeo et al., 2009), there may be unrecognized binding site variations that do not match the AW-box pattern. An attempt was therefore made to detect motifs independently of prior knowledge in a larger number of upstream regions of WRI1-coexpressed genes, using the *de novo* pattern-finding program MEME (Multiple Em for Motif Elicitation; Bailey et al., 2009). To select WRI1-coexpressed genes, we first analyzed the transcriptomic developmental time series of *A. thaliana* developing seeds that is used in Figures 1, 2. WRI1 co-expression gene sets of sizes 50, 100, 250, 500 and 1,000 were obtained when thresholds to the Pearson correlation coefficient (R) between 0.991 and 0.943 were applied. Overall, the top significant motifs MEME predicted from the associated upstream regions mainly captured motifs that can be described as A/T repeats as well as di- and tri-nucleotide repeats. Similar DNA elements were also obtained from randomly selected control gene sets. After this approach did not appear promising, we next tried to derive a network of pairwise co-expressed genes associated with WRI1. The ATTED-II co-expression database (Obayashi et al., 2007), version 11 (Obayashi et al., 2022) was mined (see Methods). Using a guide gene approach (Aoki et al., 2007) we deduced a highly interconnected WRI1-associated co-expression network with 47 *A. thaliana* genes (nodes) and 132 edges (see Methods; Supplementary Figure 3A). GO term enrichment analysis of this gene set found genes of fatty acid biosynthesis, glycolysis and acetyl-CoA biosynthesis to be highly over-represented (Supplementary Figure 3C). Analyzing the 500 bp upstream regions of the WRI1 co-expressed gene set, MEME discovered three motifs with an E-value of 1 × 10^−10^ or lower (Figure 3A). The second-ranking motif (CYTYGKTWWCWYCGHH, Figure 3A) was found in 37 out of the 47 searched upstream regions and has strong similarity to the AW-box. Searching the motif against a set of DAP-seq derived motifs (O'Malley et al., 2016), the DNA-binding profile for LBD2 (AT1G06280) had highest similarity, albeit with low significance (Figure 3A). The highest-ranking motif (MTCTCTSTYTCTCTCT) has resemblance to binding sites for proteins of BARLEY B RECOMBINANT / BASIC PENTACYSTEINE (BBR/BPC) family of transcription factors (Figure 3A). These are known to bind (CT)_n_ sequence repeats and to have function in various developmental processes (Monfared et al., 2011; Kumar and Bhatia, 2016; Theune et al., 2019). Expecting that the discovered motifs are conserved across species, we extended the set of 47 *A. thaliana* sequences by orthologs from the other *Brassicaceae* species of the study. Running MEME on the extended set of 398 sequences again identified a similar set of three top ranking motifs (Figure 3B). Motif 2 is again largely consistent with the AW-box consensus. 231 of the 243 sites that constitute the motif fully conform to the canonical AW-box [“CNTNG(N)_7_CG”] while 8 sites conform to a variant with T at the first position [“TNTNG(N)_7_CG”]. This variant will be discussed further below.

### *In vitro* binding assays of AW-box sequences

**Figure 4:**
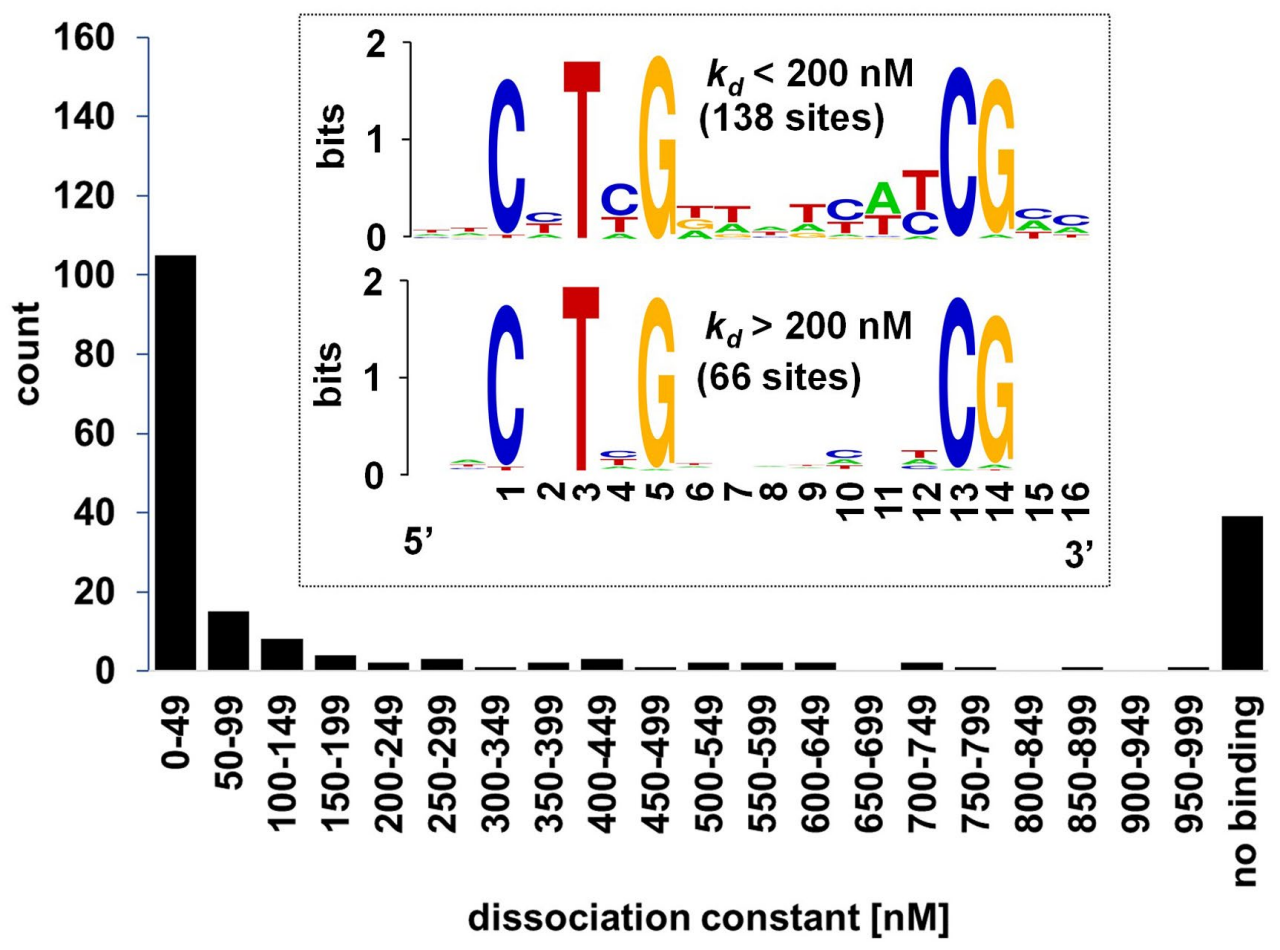
Distribution of DNA-WRI1 binding affinities for 194 DNA targets that contain the AW-box. Equilibrium dissociation constants (*k_D_*) were determined by Microscale thermophoresis (*k_D_*-values listed in Supplementary Table 4). Inset: Sequence logos of 204 AW-box sites from the measured DNA fragments, separated according to ranges of *k_D_* values. “no binding”: DNA targets classified as non-binding based on binding curve (Supplementary Figure 4). Sequence logos were generated with WebLogo (Crooks et al., 2004).

We next set out to characterize DNA-binding affinity of WRI1 protein for selected AW-sites by an *in vitro* Microscale Thermophoresis (MST) assay. The main purpose was to cover genes associated with the conversion of sucrose to TAG, for which we assembled a catalog of 309 genes (Supplementary Table 3). At the same time, characterization of a larger number of distinct AW-box sites by MST should identify a refined binding profile that specifies base bias at the “N” positions of the AW-box consensus. Sites were selected mostly upstream of genes identified by the AW-box enrichment and conservation analysis (Supplementary Tables 6, 7). Since many of these genes have both conserved and non-conserved AW-sites upstream, a roughly balanced selection of conserved and non-conserved sites was included in the analysis. As further discussed in the following sections, a few sites in upstream regions of other species as well as sites that do not fully conform to the AW-box pattern were included as well. Altogether, 194 DNA fragments upstream of 105 *A. thaliana* genes and upstream 40 genes in other species were synthesized and tested (Methods). In each case a 14 bp AW-box sequence is flanked by 6–8 bp genomic context on each side. Since AW-box sites overlap in some cases, the 194 DNA fragments encompass altogether 204 AW-box sequences. Sequence information relating to the DNA targets, MST results and other relevant details are listed in Supplementary Table 4. Figure 4 shows that the resulting *k_D_* values approximate a bimodal distribution which peaks for values close to 0 nM and for dissociation constants close to 1,000 nM. At the high end, 40 DNA targets are included that, by inspection of the binding curves, were classified as non-binding (Methods). Describing a middle section in Figure 4, *k_D_* values for 21 DNA targets are spread almost evenly between 200 and 999 nM. The left peak of the bimodal distribution is defined as the 132 DNA targets with *k_D_* values below 200 nM. When arranged in a sequence alignment, these 132 DNA targets are distinguished in that they have substantial sequence similarities while for *k_D_* values above 200 nM, essentially no conservation is apparent except for the conserved bases of the AW-box (Figure 4, sequence logos in inset). Essentially the same two sequence logos are obtained when the *k_D_* threshold is shifted by 100 nM to the left or to the right. Also, selecting binding sequences much more stringently below 10 nM, we did not obtain a much different binding profile. Overall, the result of about 200 MST assays is a binding profile that represents a significant refinement of the AW-box pattern. The base distributions at positions 2, 4, 6, 7, 9–12, 15 and 16 of the MST-derived motif (Figure 4, sequence logo for low *k_D_* values) are largely consistent with the base distributions obtained from the *de novo* motif identification (Figure 3). For other purposes below we refer to the 200 nM threshold as a threshold for strong *in vitro* binding as it delineates the left binding peak in Figure 4 and defines a consensus of *WRI1* binding sites.

At the lower end, highest affinity *k_D_* values are in the 1 to 10 nM range. Searching literature for quantitative binding data of a similar transcription factor we found that *in vitro* binding affinity has been determined before for AINTEGUMENTA, another transcription factor of the AP2/EREBP family (Nole-Wilson and Krizek, 2000). The protein was determined to bind to a 16 bp motif (5’-RCNTYGGGAWNYGTGC-3′; Nole-Wilson and Krizek, 2000), which is in part similar to the AW-box. In that study, 40 DNA fragments were sequenced after 18 rounds of *in vitro* selection and amplification of random oligonucleotides with AINTEGUMENTA protein (Systematic evolution of ligands by exponential enrichment, SELEX). One of these strongly binding sequences was analyzed quantitatively based on an electrophoretic mobility shift assay (EMSA), and a *k_D_* value of 13 nM was obtained. This value falls well within the low nM range populated by the bulk of the WRI1 binding constants determined here.

### Prediction of *in vitro* binding based on a binding consensus

**Figure 5:**
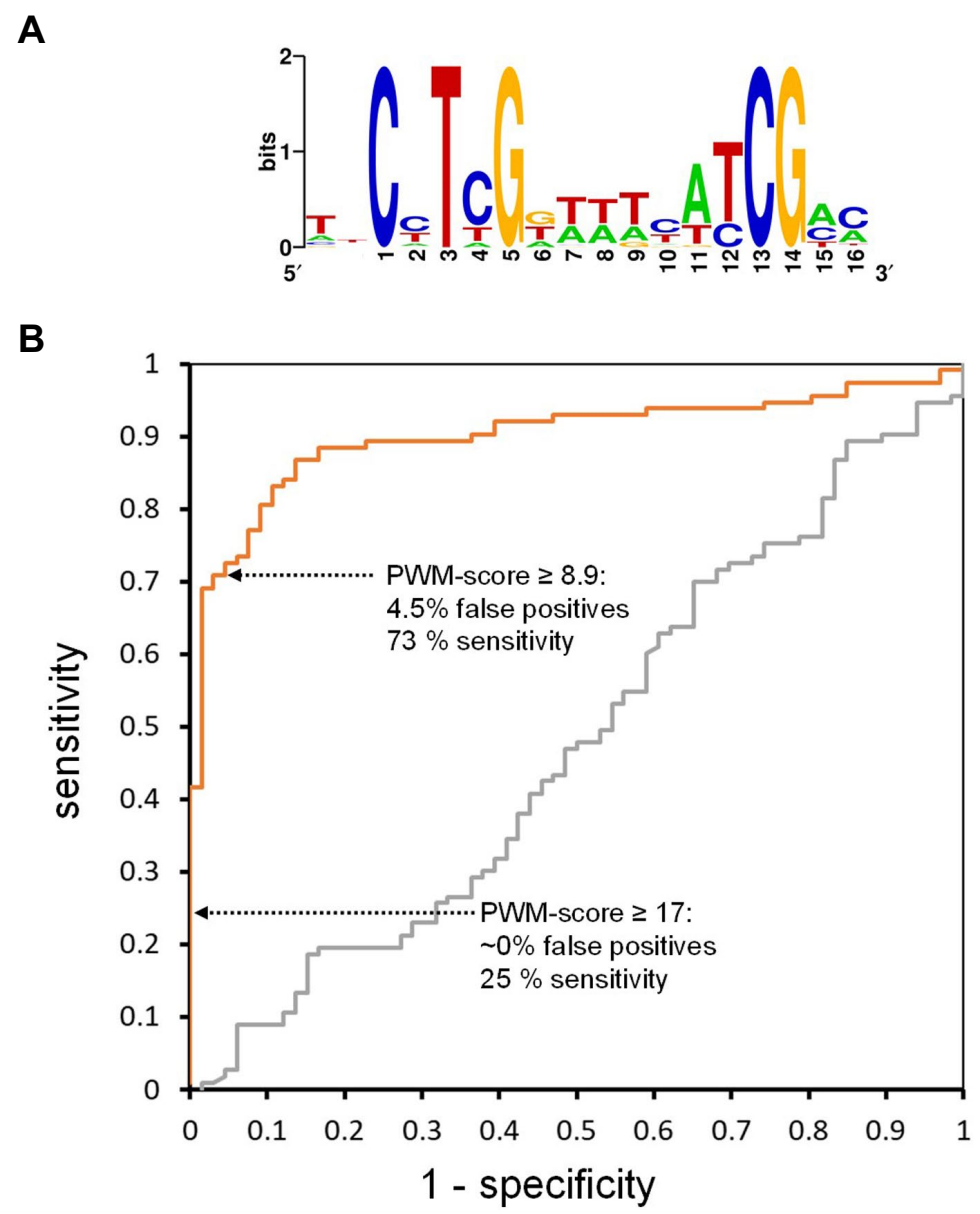
Performance of scoring matrices in binding site prediction based on a test set of target sequences with measured binding affinity. Of the set of 204 sequences for which binding to WRI1 protein was quantified by MST (Figure 4), 25 with among the highest binding affinity values were removed to define a scoring matrix (Supplementary Table 8), for which the sequence logo representation is shown in **(A)**, while the remaining 179 MST characterized sequences were used as a test set for scoring. For generation of the Receiver Operating Characteristic (ROC) curves **(B)**, 138 sequences of the test set with *k_D_* < 200 nM were considered to be truly binding. A control curve (gray) was generated after random shuffling of the 179 *k_d_* values in the test set. The area under the ROC control curve is close to 0.5 as expected for random guessing (Fawcett, 2006).

Having measured the WRI1 binding affinities for 194 DNA targets (Figure 4) we can model binding specificity. This is commonly done by representing a binding site motif as a position weight matrix (PWM), which can be used to score test sequences for similarity with the binding motif (Stormo, 2000; Wasserman and Sandelin, 2004). PWMs deduced in such a way can be used to identify candidate binding sites with sequence analysis tools like RSAT (Turatsinze et al., 2008) or FIMO (Grant et al., 2011). Since the measured binding affinities shown in Figure 4 range from high affinity binding to non-binding, we can also develop a test that uses PWM scores to classify AW-box sequences into binding and non-binding ones, based on the principles of receiver-operating characteristic (ROC) analysis (Fawcett, 2006). To do this, we divided the 204 AW-site sequences with *k_d_* measurements into a set of 25 for defining PWM and a test set of 179. The 25 were randomly chosen among 53 that had *k_D_*—values below 5 nM to define the PWM (Figure 5A, Supplementary Table 8), which was then used to calculate a PWM score for each of the test sequences (Methods). For the classification test, sequences with *k_D_* < 200 nM were considered to be truly binding and a range of PWM score thresholds were iteratively applied to classify the test sequences into binding and non-binding. In a receiver-operating characteristic (ROC) curve (Fawcett, 2006), the true positive rate (PWM score correctly predicted binding) was plotted against the false positive rate (Figure 5B). Moving along the orange curve in Figure 5B we can assess different possible PWM score classification thresholds. For example, if all test sequences with a PWM score ≥ 17 are judged to be binding, then we can expect close to 0% of the predictions to be false positive, while only 25% of the true binding sites are predicted to be binding (25% sensitivity; Figure 5B). Reducing the PWM score threshold to 8.9, we can expect 4.5% of the predictions to be false positive with 73% sensitivity (Figure 5B). Although the ROC curve procedure depends on the somewhat arbitrary *k_d_* threshold (200 nM), these characteristics do not change much if the *k_d_* threshold is shifted. For example, if we assume a 100 nM threshold to define truly binding sequences and re-create the ROC curve shown in Figures 5B, a close to 5% false positive rate and close to 75% sensitivity are obtained for a PWM scores threshold of 10. If we set a 500 nM binding threshold, a PWM score threshold of 6.5 leads again to similar characteristics. Thus, given the PWM we derived from the *in vitro* binding tests, a PWM score threshold of 10 should allow testing AW-box sites for which no *k_d_* measurements are available and we can expect an about 5% false positive rate and close to 75% sensitivity.

### AW-boxes that bind WRI1 locate close to the transcriptional start site and associated genes tend to be co-expressed with WRI1

**Figure 6:**
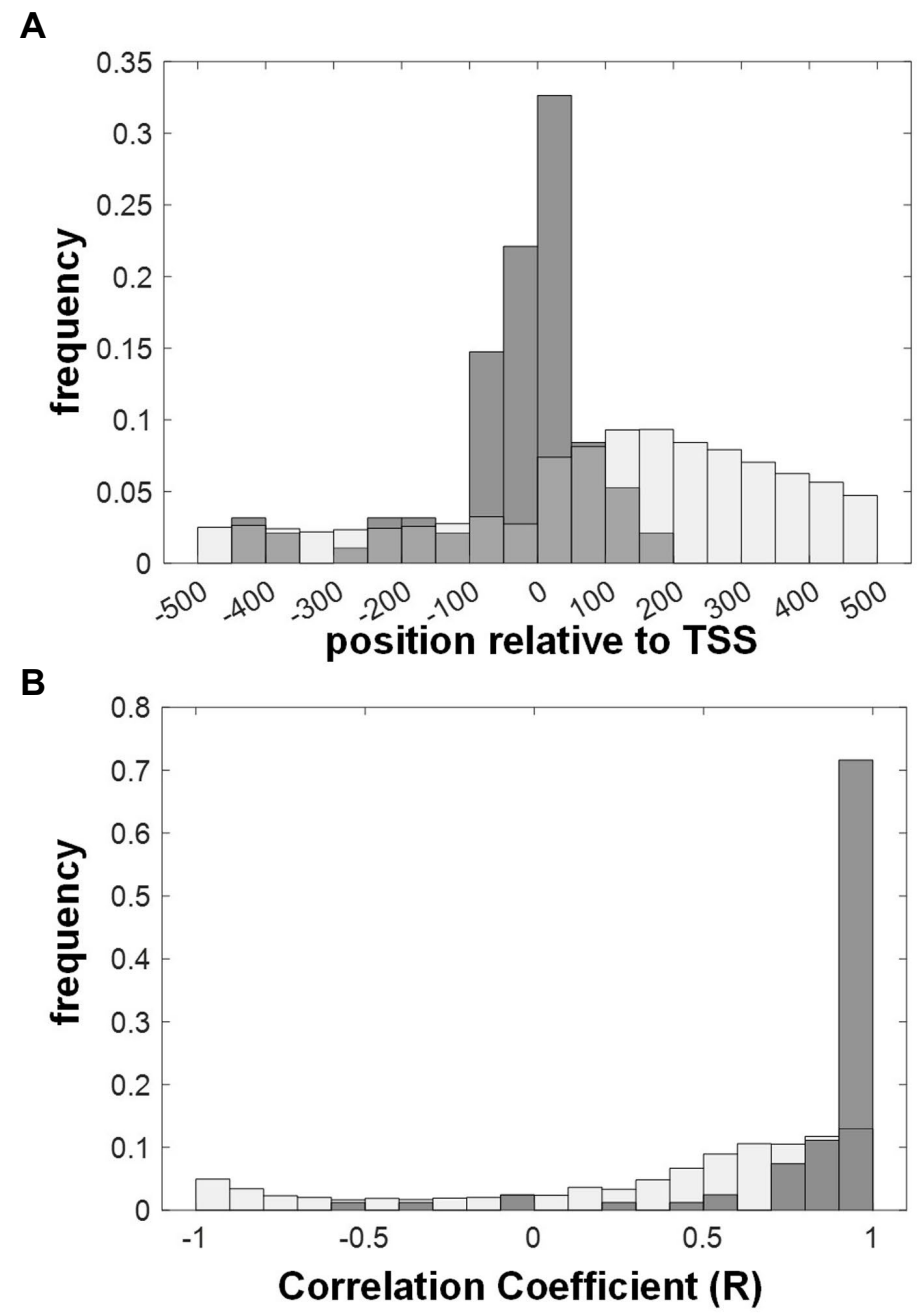
Characterization of putative WRI1 gene targets identified through *in vitro* binding AW-box sites in their upstream region. AW-box sites are classified as binding WRI1 for *k_D_* values below 200 nM (see Figure 4). **(A)** Positional distribution of WRI1 binding AW-boxes relative to the transcriptional start site (TSS) in *A. thaliana* (Gray bars). White bars: position of genome wide detected AW-sites. **(B)** Co-expression (Pearson Correlation) of *A. thaliana* genes with WRI1 during seed development. Gray bars: correlation coefficients for genes for which at least one upstream motif binds WRI1. White bars: genes with AW-box hit. Pearson Correlation coefficients are based on gene expression data for embryos in 7 seed developmental stages (Schneider et al., 2016).

It has been shown for several organisms, including *A. thaliana*, that authentic cis-regulatory elements tend to be localized in proximity of the transcriptional start site (TSS; Yu et al., 2016). Accordingly, Figure 6A shows that *A. thaliana* AW-sites that were classified to strongly bind WRI1 *in vitro* based on a *k_D_* below 200 nM (Figure 4) are positioned close to the TSS. Most of them group within a roughly 250 bp wide window around the TSS (position −100 to 150; Figure 6A). Note that the sites that were to be measured by MST were selected from a larger window (range −1 to −500 bp upstream ATG), which means that the agglomeration around the TSS is unlikely to be an effect of the selection. As a control, genome-wide occurring AW-box sites are more evenly distributed (Figure 6A). Somewhat unexpected for the genomic background distribution of AW-sites in Figure 6A is a bulge visible downstream of the TSS. The explanation for this uneven distribution is that in many cases the distance between TSS and ATG start is less than the 500 bp, which is the range shown in the figure. This means that there is a contribution of coding sequence in the positive range shown in the figure. As seen in Table 1, the AW-box occurs with about twice the density in the region downstream ATG than upstream, which in turn is explainable by the higher GC content of that region. In addition to the proximity of cis-regulatory elements to the TSS, it can be expected that genes that are regulated by the same transcription factor can be found to be co-expressed. Figure 6B shows that during seed development, the expression of gene targets for which we found strong *in vitro* binding of AW-boxes (*k_D_* < 200 nM) tends to be positively correlated with *WRI1*.

### AW-sites upstream of functionally validated WRI1 targets tend to be phylogenetically conserved

**Figure 7:**
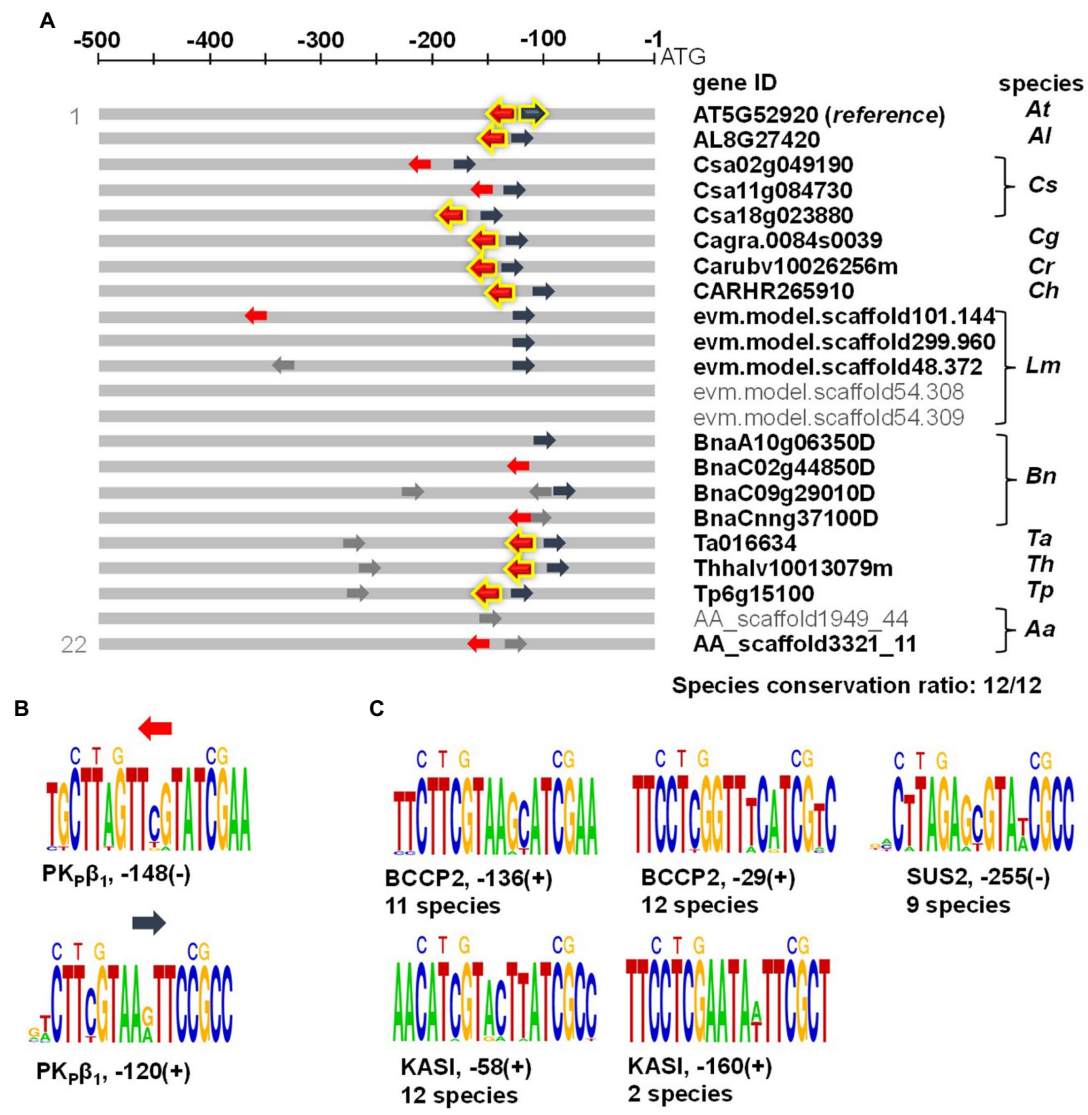
Conservation of the AW-box across 500 bp ortholog upstream regions (OURs). Sequence comparisons were made for 18 nc sequences (14 nc AW-box sites plus two nc adjacent on each side). **(A)** Each of the two AW-box sites upstream of AT5G52920 (*A. thaliana* plastidic pyruvate kinase β_1_-subunit) is compared to all other detected sites with identical directionality (+/− strand) in the OURs. The emboldened arrows represent the AW-sequence in *A. thaliana*, which in some cases are fully preserved in other species. For 15 or more base identities per 18 bases sequence length a conservation relation is given (red or dark blue color). Since pairwise conservation relations reach all the 12 species of this study, the *A. thaliana* AW-sites are considered to be conserved across all 12 species (Species conservation ratio = 12/12). **(B)** Sequence logos of the two conserved AW-box sites upstream of AT5G52920. **(C)** Sequence logos of the two conserved AW-box sites upstream of Biotin Carboxyl Carrier Protein 2 (BCCP2, AT5G15530), Ketoacyl-ACP Synthase I (KASI, AT5G46290) and sucrose synthase 2 (SUS2, AT5G49190). Number of species in which each AW site is conserved is given. All sequences shown in this figure can be found in Supplementary Table 9. Abbreviations: Aa, *Aethionema arabicum*; At, *Arabidopsis thaliana*; Al, *Arabidopsis lyrata*; Bn, *Brassica napus*; Cs, *Camelina sativa*; Cg, *Capsella grandiflora*; Cr, *Capsella rubella*; Ch, *Cardamine hirsuta*; Lm, *Lepidium meyenii*; Th, *Thellungiella halophila*; Tp, *Thellungiella parvula*; Ta, *Thlaspi arvense*.

Identification of functional cis-regulatory elements is challenging and combining diverse layers of evidence will increase the confidence in the *in vivo* functionality of target sites. Our findings suggest that genes with conserved AW-box sites in the upstream region tend to be co-expressed with WRI1 (Figure 2). We also found that AW-sites that have strong *in vitro* binding activity tend to be in proximity to the TSS (Figure 6A) and that genes with strong *in vitro* binding AW-sites tend to be co-expressed with WRI1 (Figure 6B). Taken together, the combination of conservation, proximity to the TSS, *in vitro* binding, and co-expression is likely to reliably predict WRI1 gene targets. This convergence of properties applies to 12 genes associated with FAS that have been experimentally established as WRI1 gene targets with high confidence (Table 5). Table 5 lists 8 components of the plastidic acetyl-CoA carboxylase (BADC1, BADC2, BADC3, BC, BCCP1, BCCP2, CT-α, PII), two components of the plastidic FAS complex (KASI, KASIII), as well as a component of plastidic pyruvate kinase (PK_P_-β_1_) and sucrose synthase 2. All these have previously been characterized as WRI1 targets with multiple evidence reported in 9 publications cited in Table 5. For four of them evidence of *in-planta* functionality of the AW-box as cis-regulatory element was given by Maeo et al. (2009): Two AW-sites 148 and 120 bp upstream ATG of *A. thaliana* plastidic pyruvate kinase β_1_-subunit (*At*PK_p_β_1_) have been shown to bind WRI1 *in vitro* and the ability of the two binding sites to drive *WRI1*-dependent gene expression *in-planta* was demonstrated based on reporter gene assays (Maeo et al., 2009). Here we find both sites to be localized in close proximity to the TSS, and to have binding constants well below 200 nM (Table 5). In addition, *At*PK_p_β_1_ was also found to be co-expressed with WRI1 and the two upstream sites are conserved across all species of this study (Table 5). Figure 7A shows in detail how each of the two *At*PK_p_β_1_ upstream sites is conserved across upstream regions of the 12 *Brassicaceae* species. Conservation of the *At*PK_p_β_1_ sites is missing only for three PK_p_ orthologs in polyploid species which have multiple PK_p_ orthologs (Figure 7A). While in this study we infer the conservation based on a pairwise comparison of AW sites between *A. thaliana* and other species, the result of this procedure can also be aggregated into a sequence alignment. When the conserved *At*PK_p_β_1_ AW-sites are shown as sequence logos, it is found that 13 and 14 out of 18 base positions, respectively, are fully conserved (Figure 7B). To test whether WRI1 binding activity is phylogenetically retained, we measured *k_d_* values for conserved orthologous sites of the 148 bp upstream AW-box site (Supplementary Table 9). As 8 of the 14 orthologous sites were identical to the *A. thaliana* site (Figure 7A; red emboldened arrows), binding was measured only for six other orthologous sites for which the sequence differed. *k_d_* values ranged between 0.5 and 22 nM (Supplementary Table 9). This strongly suggests that WRI1 binding activity is retained in all conserved orthologous sites. Besides the two well characterized AW-sites upstream of *At*PK_p_β_1_, Maeo et al. (2009) validated five additional WRI1 binding sites upstream of Biotin Carboxyl Carrier Protein 2 (BCCP2), Ketoacyl-ACP Synthase I (KASI) and sucrose synthase 2 (SUS2) by *in vitro* binding assays and *in-planta* by WRI1-dependent transactivation assays (Table 5). Four of the five sites are well conserved across species (Figure 7C). In case of KASI, only one of two AW-sites is well conserved among 12 species while the site 160 bp upstream is only found in two species (Figure 7C). Altogether, the 12 genes listed in Table 5 are characterized by at least 3 out of the following four conditions: less than 200 bp distance to the TSS, strong *in vitro* binding of AW-box sites to WRI1 (*k_d_* < 200 nM), conservation of AW-box sites (species conservation ratio ≥ 0.75) as well as a high correlation coefficient in the WRI1 co-expression dataset used in this study (R > 0.681, *p* value 0.05). The same selection criteria can be applied to a more complete set of 46 *A. thaliana* genes that are associated with FAS (Supplementary Table 10). For 33 of the 46 genes, AW-sites within 500 bp upstream of the ATG start codon were found. For 19 of these genes (61%) all four above criteria apply and for 29 genes (94%) only one of the above conditions is violated. The conservation selection criterion is violated most often, probably due to the complexity of the comparative analysis of genomes. For example, while in this study ortholog gene sets were derived for 12 *Brassicaceae* genomes, 27% of the ortholog gene sets contain less than 9 species. This means that for 27% of the ortholog gene sets a motif site can only be found to be conserved across less than 9 species, i.e., the species conservation ratio cannot be 0.75 or above. In addition, conserved sites present further upstream than 500 bp are not detected by our automated workflow. Among the FAS genes listed in Supplementary Table 10, conserved sites were found further upstream for Enoyl-ACP Reductase and Ketoacyl-ACP Synthase II, as documented in Supplementary Table 9. In similar, upstream AW-box sites might be missed in some cases where computational gene predictions might have missed to correctly predict the start of the first exon. Lastly, the approach used here does not completely explore synteny relations for genes that occur in tandem gene array configuration. Nevertheless, given the assessment of high confidence gene targets in Table 5 and for the extended list of genes related to FAS (Supplementary Table 10) we find that the above four selection criteria identify new gene targets with sufficient confidence to warrant further *in vivo* and *in-planta* experimental exploration. Such gene targets are listed in Table 6 and discussed in detail below.

**Table 5:** Examples of gene targets in fatty acid biosynthesis which have previously been characterized by genetic and biochemical evidence.

Gene abbreviation (description, gene ID)	Distance of binding motif to ATG start / TSS (orientation)	WRI1 dissociation constant^1^ [nM]	Species conservation ratio^2^	Gene expression correlation with WRI1^3^
*BADC***1** (Biotin/lipoyl Attachment Domain Containing, AT3G56130)^4^	−162/−53(−)	0.6 ± 0.2	0.83	0.92^**^
*BADC*2 (Biotin/lipoyl Attachment Domain Containing, AT1G52670)^4^^,^^5^	−95/+26(+)	5.7 ± 2.6	0.67	0.98^**^
*BADC*3 (Biotin/lipoyl Attachment Domain Containing, AT3G15690)^4^^,^^5^	−87/+73(+)	9.5 ± 3.1	0.58	0.94^**^
*BC* (Biotin Carboxylase, AT5G35360)^6^	−68/+62(−)	0.2 ± 0.2	0.50	0.98^**^
*BCCP***1** (Biotin Carboxyl Carrier Protein 1, AT5G16390)^7^	−60/+43(+)	62.2 ± 19.5	0.92	0.93^**^
*BCCP***2** (Biotin Carboxyl Carrier Protein 2, AT5G15530)^5^^,^^6^^,^^8^^,^^9^^–^^11^^,#^	−29/+19(+)	0.7 ± 0.3	1.00	0.99^**^
	−136/−75(+)	7.0 ± 3.5		
*CT-α* (Carboxyltransferase α-subunit, AT2G38040)^6^	−367/+9(−)	70.2 ± 9.4	1.00	0.94^**^
*KAS***I** (Ketoacyl-ACP Synthase I, AT5G46290)^5^^,^^8^^,^^11^^,^^12^^,#^	−58/+44(+)	1.7 ± 1.2	1.00	0.98^**^
	−160/−45(+)	51.1 ± 21.2		
*KAS***III** (Ketoacyl-ACP Synthase III, AT1G62640)^8^^,^^11^^,^^12^	−204/+55(+)	45.3 ± 3.8	0.83	0.96^**^
	−201/+58(−)	45.3 ± 3.8		
*PII* (PII/GLNB1 homolog, AT4G01900)^7^	−119/−45(+)	9.6 ± 4.1	1.00	0.98^**^
	−116/−42(−)	9.6 ± 4.1		
*PK*_p_*-ß*_1_ (Plastidic pyruvate kinase β_1_ subunit, AT5G52920)^5^^,^^8^^,^^9^^,^^10^^,^^11^^,^^12^^,^^#^	−120/+17(+)	2.5 ± 1.4	1.00	0.95^**^
	−148/+2(−)	12.2 ± 6.9		
*SUS***2** (Sucrose synthase 2, AT5G49190)^5^^,^^8^^,^^9^^,^^11^^,^^#^	−255/+144(−)	12.6 ± 11.5	0.75	0.75^*^

For all the listed genes, WRI1 binding curves were determined three times. For gene abbreviations listed in bold, all the following criteria apply: distance to TSS < 200 nc, WRI1 dissociation constant <200 nM, species conservation ratio ≥ 0.75, gene expression correlation coefficient significant (R > 0.681, p value 0.05). Details on the characterization can be found in the cited publications. Further details on the gene targets are shown in Supplementary Table 9.

1 *k_D_* value (mean ± SD, *n* = 3).

2 number of species in which AW-sites are conserved divided by 12 (total number of species).

3 Pearson correlation coefficients for co-expression with WRI1 from transcriptomic data sampled across 7 seed developmental stages in *A. thaliana* (Schneider et al., 2016).

4 Liu et al., 2019.

5 Ruuska et al., 2002.

6 Fukuda et al., 2013.

7 Baud et al., 2010.

8 Maeo et al., 2009.

9 Baud et al., 2007.

10 Baud et al., 2009.

11 Kim et al., 2016.

12 To et al., 2012.

* Positive correlation. Value of *p* < 0.05 for R > 0.681.

** Positive correlation. Value of *p* < 0.01 for R > 0.866.

# *in-planta* evidence for promoter functionality by reporter gene assays.

**Table 6:** Putative direct *A. thaliana* WRI1 regulatory gene targets.

Gene abbreviation (description, gene ID)	Distance of binding motif to TSS (orientation)	WRI1 dissociation constant^1^ [nM]	Species conservation ratio^2^	Gene expression correlation with WRI1^3^
*ACBP***6** (ACYL-COA-BINDING PROTEIN 6, AT1G31812)	0(+)	7.9 ± 1.0	0.92	0.97^**^
*BCA***5** (beta carbonic anhydrase 5, AT4G33580)	−89(+)	0.6 ± 0.5	1.00	0.93^**^
*bZIP*67 (Basic-leucine zipper transcription factor family protein, AT3G44460)	−3(+)	0.03 ± 0.04	1.00	0.47
*DGAT***2** (Diacylglycerol acyltransferase 2, AT3G51520)	+29(−)	39.0 ± 9.1	0.92	0.94^**^
*ENO***1** (Enolase, AT1G74030)^(p)^	−72(+)	10.3 ± 6.7	0.92	0.96^**^
*ENO***2** (Enolase, AT2G36530)^(c)^	+33(+)	13.4 ± 3.7	0.92	0.89^**^
*FRK***3** (Fructokinase, AT1G66430)^(p)^	−37(+)	85.2 ± 32.8	0.83	0.96^**^
*GPDHC***1** (glycerol-3-phosphate dehydrogenase, AT2G41540)^(c)^	+178(−)	81.7 ± 15.5	0.92	0.98^**^
*GPDH* (glycerol-3-phosphate dehydrogenase, AT3G07690)^(c)^	+34(+)	105.2 ± 30.8	0.83	0.94^**^
*LEC*1 (Leafy Cotyledon 1, AT1G21970)	+3(−)	4.3 ± 1.4	0.75	0.55
*L1L* (Leafy Cotyledon1-Like, AT5G47670)	+64(+)	10.5 ± 1.0	0.92	0.91^*^
*PFP-α* *(pyrophosphate-dependent phosphofructokinase α-subunit, AT1G76550)* ^(c)4^	−61(+)	12.3 ± 4.3	0.92	0.93^**^
*PFP-ß* (pyrophosphate-dependent phosphofructokinase ß-subunit, AT1G12000)^(c)^	+3(+)	53.9 ± 12.0	0.92	0.93^**^
*PGD***1** (6-phosphogluconate dehydrogenase, AT1G64190)^(c)(p)^	−52(+)	9.8 ± 1.2	0.83	0.96^**^
	−87(+)	70.4 ± 3.5		
*PGD***3** (6-phosphogluconate dehydrogenase, AT5G41670)^(c)(p)^	−46(+)	9.8 ± 1.2	0.67	0.91^**^
*PGI***1** (phosphoglucose isomerase, AT4G24620)^(p)^	+20(+)	2.9 ± 0.6	0.92	0.94^**^
*PGLCT* (plastidic glucose translocator, AT5G16150)	+45(−)	23.9 ± 4.7	1.00	0.82^*^
*PGLM*1 (phosphoglyceromutase, AT1G22170)^(p)^	+104(+)	1.9 ± 1.3	0.67	1.00^**^
*PGLM***2** (phosphoglyceromutase, AT1G78050)^(p)^	+78(+)	6.7 ± 4.1	1.00	0.97^**^
*PK*_p_*PK-α* (Pyruvate kinase α-subunit, AT3G22960)^(p)^	+38(+)	0.6 ± 0.3	0.92	0.97^**^
*PPT***1** (phosphoenolpyruvate/phosphate translocator, AT5G33320)	−89(−)	4.4 ± 6.6	0.83	0.93^**^
	+42(−)	6.6 ± 8.1		
*ROD***1** (REDUCED OLEATE DESATURATION 1, AT3G15820)	−46(+)	2.4 ± 0.3	0.92	0.96^**^
*TA***2** (transaldolase 2, AT5G13420)^(p)5^	−7(−)	104 ± 5.3	0.75	0.92^**^
*TKL*1 (transketolase 1, AT3G60750)^(p)^	−215(−)	1.0 ± 0.6	1.00	0.93^**^
*TPI* (Triose phosphate isomerase, AT3G55440)^(c)6^	+57(+)	76.2 ± 22.2	0.92	0.93^**^

**Figure 8:**
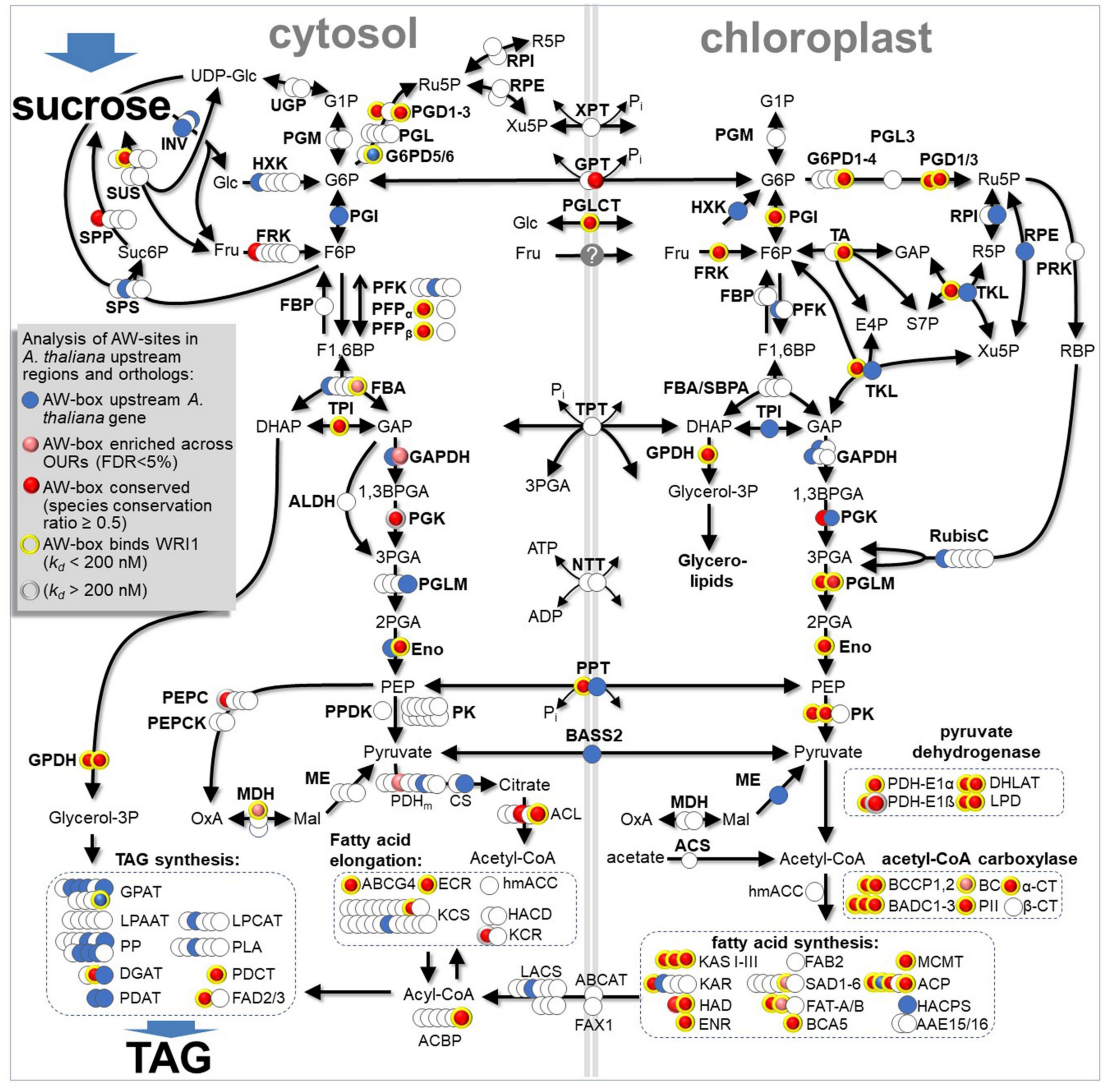
Distribution and properties of AW-box sites upstream of genes encoding for conversion of sucrose to TAG in developing seeds of *A. thaliana*. All reactions and genes are identified in Supplementary Table 3. For all genes with AW-box in the 500 bp upstream region, motif sequences, motif scores, conservation and WRI1 binding affinity are documented in Supplementary Table 9. Reaction abbreviations: AAE15/16, Acyl:acyl carrier protein synthetase; ABCAT, ABC Acyl Transporter; ABCG4, ABC Transporter (cutin, wax); ACBP, acyl-CoA-binding protein; ACL, ATP:citrate lyase; ACP, Acyl Carrier Protein; ACS, acetyl-CoA synthetase; α/β-CT, acetyl-CoA carboxylase carboxyltransferase alpha/beta subunit; ALDH, non-phosphorylating Glyceraldehyde 3-phosphate dehydrogenase; BADC, biotin/lipoyl attachment domain containing; BASS2, Sodium Bile acid symporter family protein; BC, Biotin Carboxylase of Heteromeric ACCase; BCA5, β-carbonic anhydrase 5; BCCP, Biotin Carboxyl Carrier Protein; CS, citrate synthase; DGAT, Acyl-CoA: Diacylglycerol Acyltransferase; DHLAT, Dihydrolipoamide Acetyltransferase; ECR, Enoyl-CoA Reductase; Eno, Enolase; ENR, Enoyl-ACP Reductase; FAB2, Stearoyl-ACP Desaturase; FAD2/3, oleate/linoleate desaturase; FAT, Acyl-ACP Thioesterase; FAX1, Fatty Acid Export 1; FBA, fructose bisphosphate aldolase; FRK, fructokinase; G6PD, glucose 6-phosphate dehydrogenase; GAPDH, Glyceraldehyde 3-phosphate dehydrogenase; GPAT, Glycerol-3-Phosphate Acyltransferase; GPDH, NAD-glycerol-3-phosphate dehydrogenase; HACD, Very-long-chain (3R)-3-hydroxyacyl-CoA dehydratase; HACPS, Holo-ACP Synthase; HAD, Hydroxyacyl-ACP Dehydratase; hmACC, homomeric acetyl-CoA carboxylase; HXK, hexokinase; INV, invertase; KAR, Ketoacyl-ACP Reductase; KAS, Ketoacyl-ACP Synthase; KCR, beta-ketoacyl reductase; KCS, 3-ketoacyl-CoA synthase; LACS, Long-Chain Acyl-CoA Synthetase; LPAAT, 1-acylglycerol-3-phosphate acyltransferase; LPCAT, 1-acylglycerol-3-phosphocholine Acyltransferase; LPD, Dihydrolipoamide Dehydrogenase; MDH, malate dehydrogenase; ME, malic enzyme; MCMT, Malonyl-CoA: ACP Malonyltransferase; NTT, nucleoside triphosphate transporter; PDAT, Phospholipid: Diacylglycerol Acyltransferase; PDCT, Phosphatidylcholine:diacylglycerol cholinephosphotransferase; PDH-E1α, E1-α component of Pyruvate Dehydrogenase Complex; PDH-E1ß, E1-ß component of Pyruvate Dehydrogenase Complex; PDHm, mitochondrial Pyruvate Dehydrogenase Complex; PEPC, phosphoenolpyruvate carboxylase; PEPCK, phosphoenolpyruvate carboxykinase; PFK, phosphofructokinase; PFP, pyrophosphate-dependent phosphofructokinase; PGD, 6-phosphogluconate dehydrogenase; PGI, phosphoglucose isomerase; PGK, Phosphoglycerokinase; PGL, 6-phosphogluconolactonase; PGLCT, plastidic glucose translocator; PGM, phosphoglucomutase; PGL, 6-phosphogluconolactonase; PGLM, phosphoglyceromutase; PII, regulatory subunit of acetyl-CoA carboxylase; PK, pyruvate kinase; PLA, Phospholipase A2; PP, Diacylglycerol-Pyrophosphate Phosphatase; PPDK, pyruvate orthophosphate dikinase; PPT, phosphoenolpyruvate/phosphate antiport; PRK, phosphoribulokinase; RPE, ribulose-5-phosphate-3-epimerase; RPI, ribose-5-phosphate isomerase; RubisC, ribulose bisphosphate carboxylase; SAD, Stearoyl-ACP desaturase; ß-CT, Carboxyltransferase (Subunit of Heteromeric ACCase); SPP, sucrose-6-phosphate phosphohydrolase; SPS, sucrose phosphate synthase; SUS, sucrose synthase; TA, transaldolase; TKL, transketolase; TPI, Triose phosphate isomerase; TPT, triosephosphate/phosphate antiport; UGP, UDP-glucose pyrophosphorylase; XPT, cylulose 5-phosphate / phosphate translocator. Metabolites abbreviations: 1,3BPGA, 1,3-bisphosphoglyceric acid; 2PGA, 2-phosphoglyceric acid; 3PGA, 3-phosphoglyceric acid; DHAP, dihydroxyacetone phosphate; E4P, erythrose 4-phosphate; F1,6BP, fructose 1,6 bisphosphate; F6P, fructose 6-phosphate; Fru, fructose; G1P, glucose 1-phosphate; G6P, glucose 6-phosphate; GAP, glyceraldehyde 3-phosphate; Glc, glucose; Mal, malate; PEP, phosphoenol pyruvate; R5P, ribulose 5-phosphate; RBP, ribulose bisphosphate; Ru5P, ribulose 5-phosphate; Suc6P, sucrose 6-phosphate.

For all the listed genes, WRI1 binding curves were determined three times. For gene names listed in bold all of the following criteria apply: distance to TSS < 200 nc, WRI1 dissociation constant <200 nM, species conservation ratio ≥ 0.75, gene expression correlation coefficient significant (5% level). Most of the listed genes are also shown in Figure 8. Further details on the gene targets are shown in Supplementary Table 9.

1 *k_D_* value (mean ± SD, *n* = 3).

2 number of species in which AW-sites are conserved divided by 12 (total number of species).

3 Pearson correlation coefficients for co-expression with WRI1 from transcriptomic data sampled across 7 seed developmental stages in *A. thaliana* (Schneider et al., 2016).

4 Sequence GGTTGATCGTATCG in *A. thaliana* is conserved across 9 species (non-canonical AW-motif GNTNG(N)_7_CG).

5 Sequence TCTTGGTTTGATCG in *A. thaliana* is conserved across 9 species (non-canonical AW-motif TNTNG(N)_7_CG).

6 consensus “TCTCGTGATC(A/G)TCG” is conserved across 11 species (non-canonical AW-motif TNTNG(N)_7_CG).

(c) Cytosolic compartment isoform.

(p) plastidic compartment isoform.

* Positive correlation value of *p* < 0.05 for R > 0.681.

** Positive correlation value of *p* < 0.01 for R > 0.866.

For the putative direct WRI1 gene targets listed in Table 6 we could not find literature reports on experimental characterization of upstream AW-box sites prior to this study. However, there are previous experimental findings on changes in gene expression in response to up- or down-regulation of WRI1, mostly suggesting that they are targets. One study reports global gene expression analysis for a wri1 mutant in *A. thaliana* (Ruuska et al., 2002). The study found that 41 genes varied more than approximately twofold (Supplementary Table 2 of Ruuska et al., 2002). Of these, only one gene (L1L) is among the putative new gene targets listed in Table 6. However, only 12 of the 25 genes in Table 6 are represented on the microarray of that study. Other studies have evaluated gene expression of targets listed in Table 6 under conditions of up- or downregulation of WRI1. In particular, when overexpressing WRI1 in leaves or developing seeds, expression of ENO1, PGLM1, PK_p_-α and ROD1 (Table 6) was found to be increased relative to the wild type (Baud et al., 2007, 2009; Adhikari et al., 2016). Global expression analysis with seedlings overexpressing WRI1 (Microarray) found expression of PPT1 (Table 6) to be more than 1.5-fold increased (Maeo et al., 2009). In maturing seeds of triple mutants of WRI1, WRI3 and WRI4, expression of glycerol-3-phosphate dehydrogenase isoforms GPDH and GPDHC1 and of ROD1 listed in Table 6 were reduced relative to the wild type (To et al., 2012). With regards to other species than *A. thaliana*, overexpression of a maize WRI1 homolog (ZmWri1a) in leaves and endosperm of maize increased expression of two genes encoding glycerol-3-phosphate dehydrogenase and of an isoform of beta carbonic anhydrase with predicted mitochondrial localization (Pouvreau et al., 2011)—orthologs of GPDHc1, GPDH and BCA5 in Table 6, respectively. Upon overexpression of WRI1 homologs of several species in *Nicotiana benthamiana* leaves and wheat endosperm, increase in expression was found for homologs of ENO1/ENO2, GPDHC, PGLM1/PGLM2, PK_p_-α, PPT1 and ROD1 (Grimberg et al., 2015, 2020). Potato homologs of ACBP6, ENO1, FRK3, PGI1, PGLM1, ROD1 and PPT1 (Table 6) were upregulated in potato tubers expressing *At*WRI1 (Hofvander et al., 2016). Overall, 7 genes listed in Table 6, most related to glycolysis, were reported to have a measurable transcriptional response to WRI1 overexpression or repression in Arabidopsis. For 4 additional genes the same could be shown for orthologs in other species. Also, former studies evaluating enzyme activities in wri1 mutants of *A. thaliana* found substantial reductions in activities of the glycolytic enzymes Fructokinase, pyrophosphate-dependent phosphofructokinase, phosphoglycerate mutase and enolase (Focks and Benning, 1998), for which genes are listed in Table 6. *In situ* enzyme assays on developing embryos showed strong reduction in enzyme activity for fructokinase, pyrophosphate-dependent phosphofructokinase and phosphoglycerate mutase (Table 6) in developing wri1 embryos relative to WT (Baud and Graham, 2006).

## Discussion

The transcription factor WRI1 has been described as a positive master regulator of lipid accumulation in developing seeds with several gene targets in fatty acid biosynthesis as well as late steps in glycolysis (Cernac and Benning, 2004; Baud and Lepiniec, 2009; Kong and Ma, 2018). To uncover more about the role of WRI1 in controlling the entire process of sucrose to TAG conversion during seed development, we set out to identify additional putative WRI1 gene targets for future detailed experimental characterization. Prediction of cis-regulatory elements based on motif searches in promoter regions is known to be challenging mainly because most binding sites are short and variable, predisposing to high false positive rates (Wasserman and Sandelin, 2004). As exemplified in Table 1, this limitation certainly applies to the AW-box motif, which is defined by only five conserved bases.

In view of this challenge, combining motif pattern searches with different layers of evidence, such as co-expression information or motif conservation, has proven to be a useful strategy (Bulyk, 2003; Rombauts et al., 2003; Lindemose et al., 2014). In this study we developed and tested such an approach for WRI1. To do this we explored potential binding sites first genome wide and with a phylogenetic footprinting approach (Figures 1, 2). While determining binding affinity of WRI1 to about 200 selected DNA targets that are mostly associated with central metabolism, we could also refine the consensus for WRI1 DNA binding. To identify putative gene targets for future *in vivo* and *in-planta* experimental exploration, we combine information about DNA-binding affinity, binding site proximity to the TSS and binding site conservation with co-expression information. Multiple lines of evidence support the utility of the approach: (1) Our approach identified a number of expected high-confidence WRI1 gene targets for which multiple lines of evidence have been published, including *in-planta* transactivation assays with reporter genes (Table 5). (2) As discussed below, several identified gene targets listed in Tables 5, 6; Supplementary Table 9 are found along contiguous stretches of FAS and other metabolic pathways, which is unlikely to be coincidental but rather indicative of a concerted control over multiple steps in a pathway. (3) As further detailed below, the description of seed phenotypes in previously published *A. thaliana* genetic studies strongly supports the validity of two of the newly predicted gene targets, the plastidic isoforms of fructokinase (*FRK*3) and phosphoglucose isomerase (*PGI*1). (4) We find strong *in vitro* binding AW-sites in *A. thaliana* to be located closely to the TSS (Figure 6A), consistent with findings by Fukuda et al. (2013), showing that the *in vivo* functionality of AW-box sites upstream of BC and CT-α is highly dependent on their proximity to the TSS. (5) Most of the genes associated with the strong *in vitro* binding AW-sites are co-expressed with *WRI1* during seed development (Figure 6B).

Based on the *in vitro* MST binding assay, we were able to determine equilibrium dissociation constants for WRI1 interacting with approximately 200 DNA targets representative of selected AW-box sites. This resulted in a wide range of *k_D_* values as well as 40 AW-box sites that do not bind in our assay (Figure 4). If we consider the genes listed in Table 5 to be true WRI1 targets with high confidence, then it is striking that the *k_D_* values range between 0.2 and 70 nM. It is tempting to speculate that *in vitro* binding strength might have a major impact on the strength of gene expression observable *in vivo*. While we have not further explored this possibility here, it needs to be considered that *in vivo* transcription factor binding and activity in eukaryotes can be strongly influenced by factors such as chromatin state and interaction with other transcription factors, in addition to binding sequence affinity (Slattery et al., 2014).

Based on the *in vitro* MST binding assay we could derive a binding motif (Figure 4) that agrees with the AW-box consensus and is largely in agreement with motifs found by the MEME *de novo* motif finding approach applied to upstream sequences of WRI1 co-expressed genes (Figure 3). Recently, by exploration of AW-box sites upstream of FAS genes in sunflower, a very similar motif was described for the WRI1 homolog in sunflower (Sánchez et al., 2022). During this investigation, we also made limited exploration of AW-box binding site variants that might occur less frequently than the canonical ones. For several genes involved in lipid metabolism, enoyl CoA reductase (ENR), GLNB1 homolog (PII), 3-ketoacyl-acyl carrier protein synthase II and II (KASII, KASIII), and plastidic pyruvate dehydrogenase E2 subunit (PDH E2), there are AW-sites with overlapping upstream positions, as documented in Supplementary Table 9. Overall, these overlapping sites conform to the pattern “CNTCG(N)_7_CGANG,” which hides two AW-box sites and is palindromic since it is identical to its reverse complementary translation. The full pattern occurs with partial conservation across species. Besides the palindromic variation, the sequence “GGTTGATCGTATCG” was found upstream of the α-subunit of the pyrophosphate-dependent phosphofructokinase (PFP-α, AT1G76550; Table 6). Here, cytosine at position one of the AW-box consensus is replaced with guanine and the sequence is fully conserved in 9 species (Supplementary Table 9). In terms of *in vitro* binding, distance to the TSS, species conservation and co-expression to WRI1 this non-canonical site qualifies as a likely true target site (Table 6). In addition, we found cases where cytosine at position one of the AW-consensus is replaced by thymine. For example, upstream of triose phosphate isomerase (TPI), the sequence “TCTCGTGATC(A/G)TCG” is conserved across 11 species (Table 6). In case of transaldolase 2 (TA2), a conserved site is described by the pattern “TCTTGGTTTGATCG” (Table 6). A third cases of this variant with thymine at position one of the AW-box found upstream of the pyruvate dehydrogenase E1-α subunit (AT1G01090) is well conserved and binds with a dissociation constant of 10.8 nM (Supplementary Table 9). The AW-box variant with thymine at position one was also found by the *de novo* motif discovery approach in Figure 3.

A main motivation of this study was to find out how WRI1, as a global regulator, coordinates enzyme steps involved in the conversion of sucrose to TAG. Below we highlight some candidate gene targets. Table 6 summarizes 25 potential gene targets most of which relate to the conversion of sucrose to TAG. For 21 of the 26 genes listed, all of the four selection criteria for high probability WRI1 targets apply (Table 6, gene names in bold). In Figure 8, findings on AW-box site conservation and *in vitro* binding are mapped onto a pathway scheme for the conversion of sucrose to TAG. Evidence for AW-box site conservation and WRI1 binding affinity is found for about 30 genes in three major protein complexes involved in the conversion of pyruvate into fatty acids, the chloroplast pyruvate dehydrogenase complex, acetyl-CoA carboxylase and fatty acid synthesis (Figure 8). While most of these are canonical components of FAS, β-Carbonic Anhydrase 5 (*BCA*5) is identified as an additional likely WRI1 target (Table 6; Figure 8). *AtBCA*5 has been shown to be a carbonic anhydrase isoform that localizes to the chloroplast (Fabre et al., 2007) where the enzyme might be required when FAS operates at high rates for conversion of CO_2_ to bicarbonate (HCO_3_^−^). This is because within the fatty acid synthesis process, acetyl-CoA carboxylase (ACC) and ketoacyl-ACP synthase (KAS) create a cycle of carboxylation and decarboxylation where ACC requires bicarbonate (HCO_3_^−^) as a substrate (Li-Beisson et al., 2013) while KAS (EC. 2.3.1.41) releases CO_2_. In support of a requirement for carbonic anhydrase to turn CO_2_ into HCO_3_^−^ while FAS is operating at high rates, specific BCA inhibitors have been shown to inhibit FAS in developing embryos of cotton (*Gossypium hirsutum*; Hoang and Chapman, 2002).

Like the concerted control of most genes encoding FAS enzymes by WRI1, a contiguous lower section of glycolysis is recognized as being controlled by WRI1 (Figure 8): the plastidic phosphoglycerate mutase (*PGLM*), plastidic and cytosolic enolase (ENO), the phosphoenolpyruvate/phosphate translocator (*PPT*; Table 6) as well as the α- and β-subunits of plastidic pyruvate kinase (PK; Table 5). This set of enzymes and transport functions allows phosphoenolpyruvate generated in either the cytosol or the plastid to be converted into pyruvate in the plastid. Another coherent pathway section can be recognized in α- and β-subunits of pyrophosphate-dependent phosphofructokinase (PFP), the cytosolic isoforms of fructose bisphosphate aldolase (FBA), triose phosphate isomerase (TPI) and NAD- glycerol-3-phosphate dehydrogenase (GPDH; Figure 8; Table 6). This set of enzymes can be understood as a module for the conversion of fructose 6-phosphate into the TAG precursor glycerol-3-phosphate in the cytosol.

The entry of maternally supplied sucrose into seed metabolism requires sucrose to be cleaved by invertase or sucrose synthase. In either case fructose is obtained and must be transformed by fructokinase (FRK) into fructose 6-phosphate (F6P; Figure 8). Among 6 cytosolic and one plastidic isoform for FRK, the chloroplast localized *FRK*3 was identified as a likely WRI1 target (Table 6; chloroplast localized FRK in Figure 8). While this activity makes sense in the context of converting sucrose to TAG, it is unclear how fructose would get into the chloroplast in the first place. Nevertheless, based on a recent complete biochemical and genetic characterization of the FRK gene family in *A. thaliana* (Stein et al., 2016; Riggs et al., 2017) there is support for *FRK*3 having function in the TAG biosynthesis process. While no severe seed phenotype was found for the single-KO mutation of *FRK*3, a *FRK*1-*FRK*3 double-KO mutation resulted in a severe wrinkled seed phenotype with strong reduction in seed oil content (Stein et al., 2016), providing genetic evidence that seed oil synthesis depends predominantly, although not exclusively, on contributions of this isoform. Furthermore, in mutants of WRI1 (*wri1-1*, *wri1-2*), fructokinase enzyme activity was found to be reduced by approximately 40% during seed development compared to wild-type (Focks and Benning, 1998). *In situ* enzymatic assays on developing embryos showed strong reduction in fructokinase activity in *wri1-1* as well (Baud and Graham, 2006). Besides fructokinase, it is notable that hexokinase activity (glucose as substrate) was reduced as well by about 80% in developing seeds of *wri1-1* and *wri1-2* (Focks and Benning, 1998) and almost entirely in the *in situ* assays in *wri1-1* embryos (Baud and Graham, 2006). According to our analysis, among 6 hexokinase isoforms in *A. thaliana* the most likely candidate to be a WRI1 target is the plastidic isoform HXK3 (AT1G47840). We have identified an AW-box site upstream of this gene which is conserved in four of 12 species of this study (Supplementary Table 7). However, this AW-site was not among the ones for which WRI1 binding affinity was measured and further investigation is needed to clarify whether HXK3 is a likely target. In addition to FRK3 we identified plastidic phosphoglucose isomerase (*PGI*1) as a putative WRI1 target (Table 6; Figure 8). As in the case of *FRK*3, there is recent genetic evidence in support of PGI1 being important for TAG synthesis. A *PGI*1 mutant (*pgi1-2*) was reported to have reduced seed yield per plant, seed size and seed oil content (Bahaji et al., 2018). Reciprocal crosses of *pgi1-2* with wild type showed that the low oil, wrinkled seed phenotype is independent of maternal influences (Bahaji et al., 2018). This strongly corroborates our prediction of *PGI*1 being a WRI1 target and thus important for oil synthesis. Both likely new WRI1 targets, *FRK*3 and *PGI*1, have in common that they are associated with upper glycolysis.

Additional putative WRI1 targets relate to the OPPP, which is considered a major source of reductant in heterotrophic plant tissues, delivering NADPH for various biosynthetic processes (Neuhaus and Emes, 2000; Kruger and von Schaewen, 2003), including FAS (Neuhaus and Emes, 2000; Rawsthorne, 2002). We identified several OPPP-associated genes as likely WRI1 targets (see Table 6; Figure 8): Transketolase (*TKL*1), transaldolase (*TA*2), and two isoforms of 6-phosphogluconate dehydrogenase (*PGD*3 and *PGD*1), the two of which accumulate both in the cytosol and the chloroplast (Holscher et al., 2016). Based to the GenomicusPlants web resource (Louis et al., 2015), *PGD*1 and *PGD*3 result from a whole genome duplication event at the basis of the *Brassicaceae*. One AW-box site appears to be conserved for both genes (Supplementary Table 9) and is also found more widely conserved across dicot species (Supplementary Figure 8). WRI1 binding assays also implicate functional AW-sites for isoforms of glucose 6-phosphate dehydrogenase (*G6PD*4), but less well conserved. However, this finding is somewhat ambiguous as this AW site is shared with ATP:Citrate lyase, subunit A (ACLA-3). *G6PD*4 and ACLA-3 are direct neighbors on chromosome 1 in a head-to-head configuration with 419 bp between the transcriptional start sites.

In prior studies on metabolism of seed oil synthesis a general understanding emerged that glycolysis likely takes place in both the cytosolic and the plastid subcellular compartments (White et al., 2000; Rawsthorne, 2002; Ruuska et al., 2002). Exchange of glycolytic intermediates is thought to take place at the level of phosphoenolpyruvate (PPT, Table 6; Figure 8), but also at the level of glucose 6-phosphate *via* the glucose-6-phosphate/phosphate translocator (GPT). We find AW-box conservation for GPT2 (Figure 8), but the AW-box site for *A. thaliana* and some of the orthologs did not bind in the MST assay (see Supplementary Table 9). While the results for the GPT are not clear we found, more unexpected, a conserved AW-box in the promoter of the plastidic glucose translocator (*PGLCT*; Weber et al., 2000; Table 6; Figure 8). Isolated chloroplasts of *B. napus* developing embryos have been shown to have the ability to take up glucose with saturation kinetics consistent with transporter facilitated uptake (Eastmond and Rawsthorne, 1998). Moreover, the uptake capacity of embryo chloroplasts was substantially higher than that of isolated leaf chloroplasts (Eastmond and Rawsthorne, 1998). It is currently unclear what role this transporter could have in a heterotrophic context in seed development during oil accumulation. The *PGLCT* has been implicated to be involved in photo-assimilate export from leaf chloroplasts when starch mobilization takes place in the dark (Cho et al., 2011). One study suggests that, while the bulk of starch degradation products might be exported from the chloroplast in the form of maltose, PGLCT might influence the stromal concentration of glucose and thereby be involved in the control of starch degradation (Li et al., 2017). An involvement of PGLCT in the control of starch turnover during seed development (Andriotis et al., 2010) might therefore be possible.

Although TAG biosynthesis has not previously been described as being under control of WRI1, our data identify several candidates for direct WRI1 targeting associated with the TAG biosynthetic sub-network (Figure 8): These include two isoforms of cytosolic glycerol 3-phosphate dehydrogenase (GPDH), Acyl-CoA binding protein 6 (*ACBP*6), Diacylglycerol acyltransferase 2 (*DGAT*2) and *REDUCED OLEATE DESATURATION*1 (*ROD*1; Table 6; Figure 8). Of the three different and functionally non-redundant types of DGAT found in plants, *DGAT*1 has been suggested to be responsible for most of the TAG synthesis during seed development (Li-Beisson et al., 2013). However, our results suggest that *DGAT*2 is under direct control of WRI1 (Table 6). In contrast to *DGAT*1, *AtDGAT*2 has been reported to have preference for 18:3-CoA relative to other fatty acid CoA esters (Zhou et al., 2013), which could point to a contribution of *DGAT*2 for channeling polyunsaturated fatty acids into TAG. One of the other genes putatively under direct control of WRI1, *ROD*1, encodes for phosphatidylcholine:diacylglycerol cholinephosphotransferase (PDCT) and has also been implicated in regulating the poly-unsaturation state of TAG (Lu et al., 2009). Consistent with WRI1 transcriptionally activating *ROD*1, *ROD*1 expression is significantly increased when *WRI1* is overexpressed in *A. thaliana* developing seeds (Adhikari et al., 2016) or in *Nicotiana benthamiana* leaves (Grimberg et al., 2015). In addition, *ROD*1 expression was found to be reduced in the *wri1 wri3 wri4* triple mutant in *A. thaliana* (To et al., 2012). Another TAG biosynthetic enzyme putatively under direct control of WRI1 and involved in TAG synthesis is Glycerol 3-phosphate dehydrogenase, which provides the glycerol backbone for TAG. In *A. thaliana*, two cytosolic isoforms (AT2G41540, AT3G07690) have been identified (Shen et al., 2006) as well as a plastidic one AT5G40610 (Wei et al., 2001). Notably, we have found indications for control by WRI1 for all three genes (Figure 8).

The transcription factor WRI1 is positioned toward the end of a gene regulatory cascade governing seed development and storage accumulation (Lepiniec et al., 2018). WRI1 is likely under direct control of *LEAFY COTYLEDON*1 (*LEC*1), *LEAFY COTYLEDON*2 (*LEC*2) and *FUSCA*3 (*FUS*3; Fatihi et al., 2016). Data presented herein suggests that WRI1 directly controls its own regulator, *LEC1* (Table 6), implying a condition of positive or negative autoregulation. In addition, *LEAFY COTYLEDON1-LIKE* (*L1L*) as well as the basic leucine zipper transcription factor 67 (*bZIP*67) were also identified as putative WRI1 targets (Table 6). L1L is known to act in association with *bZIP*67 in transcriptional activation of several genes related to seed storage accumulation (Yamamoto et al., 2009; Mendes et al., 2013), including cruciferin 3 (*CRU*3) and *SUS*2 (Yamamoto et al., 2009), a well-established WRI1 target (Table 5).

## Conclusion

In this work we demonstrate that a workflow based on the identification of phylogenetically conserved binding sites along with quantitative *in vitro* binding assays can be a powerful approach to identify potential regulatory networks. In addition to various known WRI1 targets associated with oil synthesis, our study revealed several other candidate genes that warrant further investigation. Mapping these putative gene targets onto central metabolism (Figure 8) will help to better understand the orchestration of TAG biosynthesis by WRI1. Besides the focus of this work on WRI1, our phylogenetic approach presented herein can be generally applied to other TFs and expanded to other plant families.

Our study relies on the assumption that cis-regulatory binding sites tend to be phylogenetically conserved across related species. While this study was mostly limited to the *Brassicaceae* family, we provide exploratory examples of AW-sites being deeply conserved in other plant families (Supplementary Figures 6–8). The phylogenetic approach should be particularly justifiable in the context of the likely highly preserved central metabolism and lipid metabolism metabolic networks. However, in addition to the gene functions discussed here, there might be additional targets of WRI1 outside the context of seed oil biosynthesis and seed development (Kong et al., 2017; Liu et al., 2019). It is possible that WRI1 binding sites upstream of other gene targets are functional but less evolutionary preserved. In the future it might be important to explore WRI1 gene targets more globally since WRI1 has been widely used in efforts at metabolic engineering of TAG synthesis in vegetative tissues of bioenergy crops (Xu and Shanklin, 2016). In this context it has been reported that strong expression of WRI1 in leaf tissue can have toxic effects and perturb vegetative development (Marchive et al., 2014; Yang et al., 2015). Such effects might be due to currently unrecognized targets of WRI1 and roles of this transcription factor during the plant’s life cycle outside of seed development. Another aspect not explored in our study is the functional overlap of WRI1 with closely related transcription factors *WRINKLED3* and *WRINKLED4*. It has been shown that *A. thaliana WRINKLED*4 regulates cuticular wax biosynthesis and has many gene targets in common with WRI1 (Park et al., 2016). Since Figure 8 shows three WRI1 gene targets related to wax/cutin synthesis it would be interesting to use this approach to evaluate *WRINKLED*4 gene targets and investigate its regulatory overlap with WRI1.

## Data availability statement

Genomic information used for data analyses was obtained from publicly available sources as listed in Supplementary Table 1.

## Author contributions

JSc, SM, CK, JK, and JSh conceived the original research plans and designed the experiments. CK, SM, and JSc performed the research and analyzed the data. SM and JSc designed and performed computational analysis. All authors contributed to the article and approved the submitted version.

## Funding

This work was supported by the U.S. Department of Energy, Office of Science, Office of Basic Energy Sciences under contract numbers DE-SC0012704 (to JSc) and KC0304000 (to JSh)—specifically through the Physical Biosciences program of the Chemical Sciences, Geosciences and Biosciences Division.

## Conflict of interest

The authors declare that the research was conducted in the absence of any commercial or financial relationships that could be construed as a potential conflict of interest.

## Publisher’s note

All claims expressed in this article are solely those of the authors and do not necessarily represent those of their affiliated organizations, or those of the publisher, the editors and the reviewers. Any product that may be evaluated in this article, or claim that may be made by its manufacturer, is not guaranteed or endorsed by the publisher.

## Footnotes

^1^ http://www.microsoft.com

^2^http://aralip.plantbiology.msu.edu/data/aralip_data.xlsx,

^3^ www.ncbi.nlm.nih.gov

^4^ http://www.bioinformatics.org/sms2/dna_pattern.html

^5^ http://weblogo.berkeley.edu/

^6^ https://david.ncifcrf.gov/

^7^ https://atted.jp/

^8^ https://nanotempertech.com/
