# Supplementary material for: An expanded role for the transcription factor WRINKLED1 in the biosynthesis of triacylglycerols during seed development
Source: Front Plant Sci. 2022 Aug 5;13:955589. doi: 10.3389/fpls.2022.955589 (PMC9389262; doi:10.3389/fpls.2022.955589)
Supplement: Supplementary file 1 [file Data_Sheet_1.PDF]

Supplementary Figures

Cathleen Kuczynski, Sean McCorkle, Jantana Keereetaweep, John Shanklin, Jorg Schwender: An Expanded Role for the Transcription Factor WRINKLED1 in the Biosynthesis of Triacylglycerols during Seed Development.

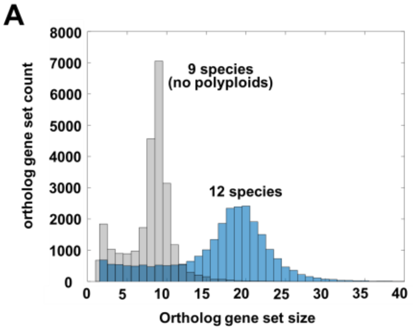

B

species	Fraction of ortholog gene sets with a contribution of this species (%)	Average number of genes contributed to ortholog gene set
Aethionema arabicum*	63.88	1.77
Arabidopsis lyrata	84.27	1.05
Brassica napus*	89.37	4.17
Camelina sativa*	88.67	2.75
Capsella grandiflora	75.81	1.04
Capsella rubella	78.93	1.04
Cardamine hirsuta	77.78	1.11
Lepidium meyenii*	74.11	3.66
Thellungiella halophila	74.48	1.05
Thellungiella parvula	72.39	1.03
Thlaspi arvense	68.98	1.09
	77.15 (average)	19.76 (sum)

*polyploid species

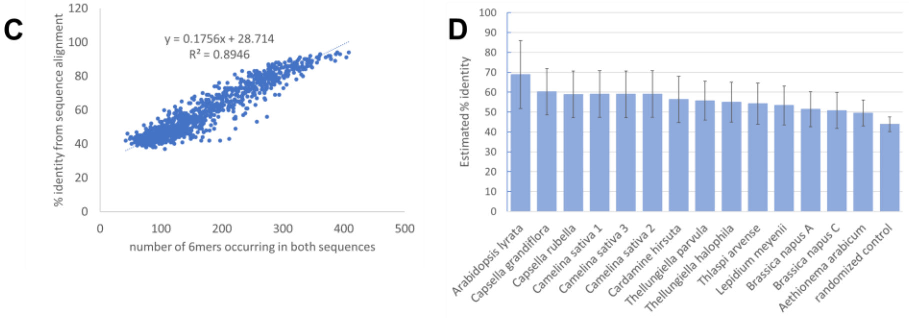

Supplementary Figure 1. Synteny/Orthology relations and sequence similarity in genomic upstream regions across *Brassicaceae* species used in this study. A, size distribution of sets of orthologous genes after synteny analysis. The SynOrths tool (Cheng et al. Front Plant Sci 2012, 3: 198) was used. 399797 pairwise orthology relations were obtained between genes of *A. thaliana* and 11 Brassicaceae species, which were aggregated into 25545 ortholog gene sets (blue bars). While there are 11 pairwise species comparisons, the median gene set size is 18. If only genome alignments between *A. thaliana* and 7 other diploid species are considered, the median gene set size is 9. The large median gene set size for the alignment of all 12 species might be explainable by the polyploidy in some of the species. This was tested in B. B, in average, each *A. thaliana* gene relates to close to one gene in the 7 diploid species, but to 1.77, 4.17, 2.75, and 3.66 genes in *Aethionema arabicum*, *B. napus, C. sativa*, and *L. meyenii*, respectively. The multiple orthologs can be explained by the polyploidy of the latter species (main text). C, Estimation of % identity between orthologous 500 bp DNA sequences based on alignment free comparison of 6mers. For 1000 random selected pairs of orthologous 500 bp upstream regions (500 pairs *A. thaliana*/*A. lyrata*, 500 pairs *A. thaliana*/*Aethionema arabicum*), the number of 6mers that occur in both sequences was determined. Then pairwise sequence alignments were generated with Clustal W 2 (ver. 2.1) (Larkin et al. Bioinformatics 2007, 23, 2947-2948. https://doi.org/10.1093/bioinformatics/btm404). The linear trend shown in panel C was used to convert 6mer counts into % identity. D, Average sequence similarity (with standard deviation) between 500 bp upstream regions of *A. thaliana* protein encoding genes and orthologous upstream regions in all compared species. The sequence comparison was done as alignment-free comparison (panel C). “Randomized control” is a re-run of the analysis for *A. lyrata* after random perturbation of the orthology relations.

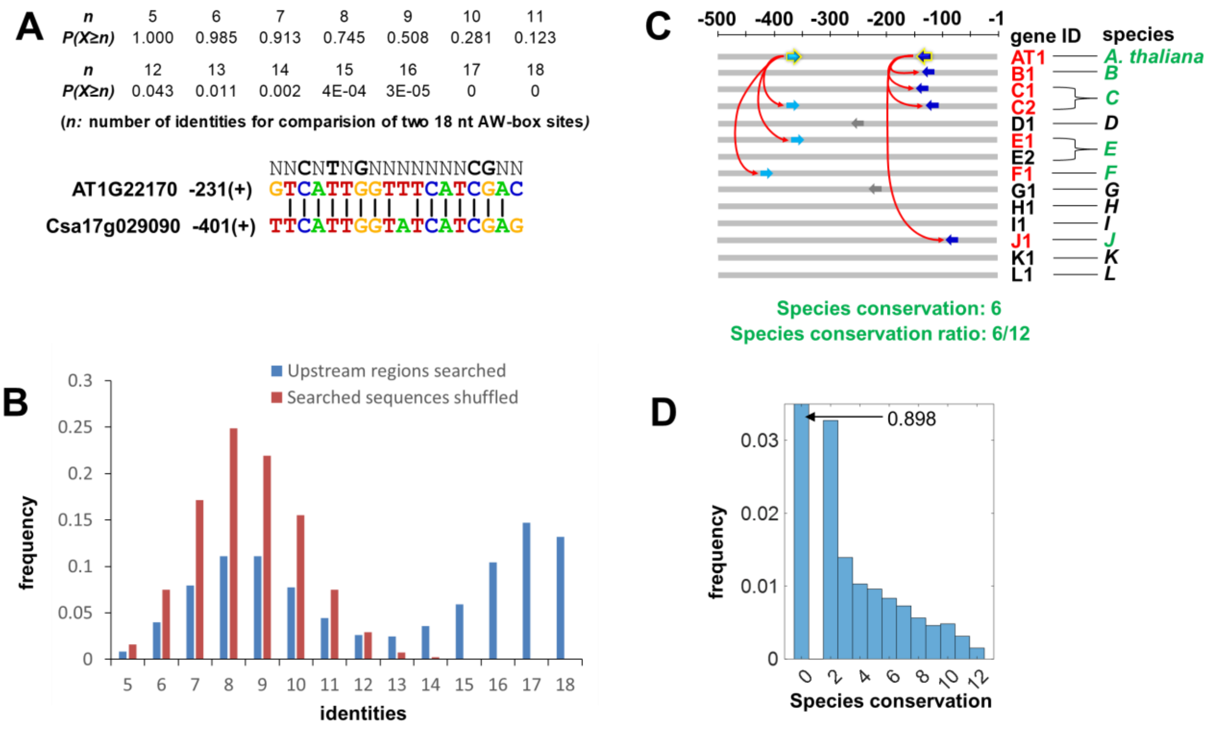

Supplementary Figure 2. Conservation of AW-box sites between *Arabidopsis thaliana* and other species. **(A)** Cumulative base identity probabilities for comparison of random sampled AW-box sites (GC content 33.2 %). An example for a pairwise alignment with 15 identities is shown. **(B)** Base identity between *A. thaliana* AW-box sites in the 500 bp upstream region and AW sites in orthologous regions. As a control, the searched sequences were shuffled. For the unperturbed sequences, there were altogether 6641 *A. thaliana* AW-box sites but in 1379 cases there was no orthologous site in same DNA strand orientation to compare with (*i.e.* 5262 comparisons are made). While identities above 14 occur frequently for comparison of genomic AW sites, they are almost absent in the random control (Panel A) as well as for the permutation control (shuffled sequences). We define that two sites with identical directionality (+/- strand) are judged to be conserved for 15 or more identities per 18 bases sequence length. **(C)** Illustrative example for the conservation analysis of an ortholog gene set. Analysis of sequence conservation is restricted to pairwise comparisons between all *A. thaliana* AW-box sites and orthologous sites in other species. Conservation relations between AW-boxes define gene level conservation. For species level conservation, one gene level conservation relation (e.g. AT1, E1) is sufficient to define a species conservation relation. In the example, the AW-box is recognized to be conserved in upstream regions of 7 out of 14 genes (red) and in 6 out of the 12 species of the study (green). **(D)** Species conservation of AW-box sites for all *A. thaliana* genes with orthologs.

A

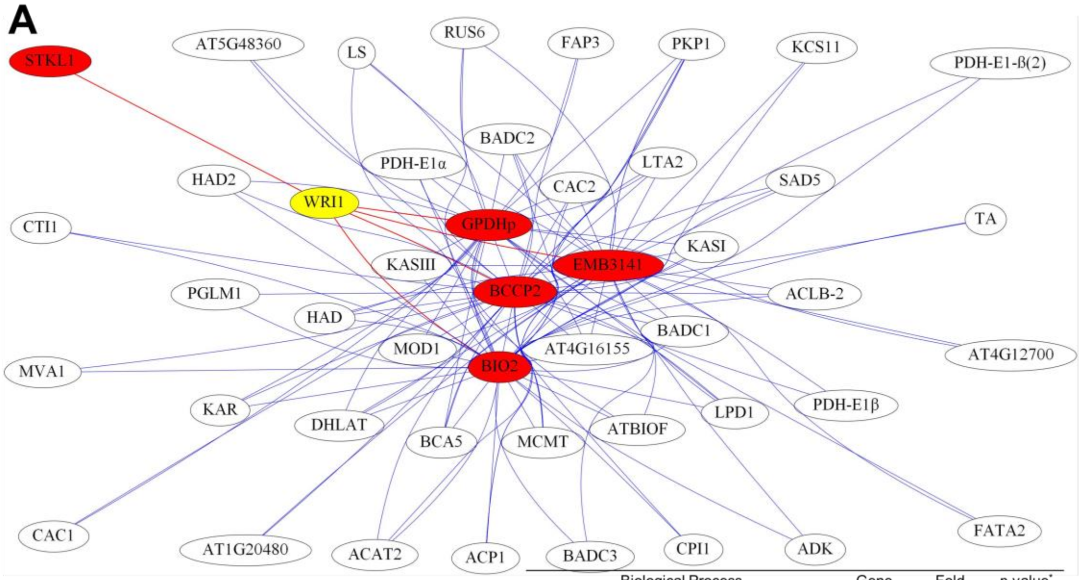

B

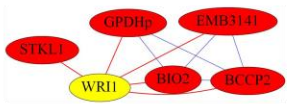

Gene	connectivity
STKL1	1
BIO2	38
EMB3141	34
BCCP2	36
GPDHp	28

C

Biological Process	Gene count	Fold Enrichment	*p*-value*
fatty acid biosynthetic process (GO:0006633)	19	76.43	1.74E-28
glycolytic process (GO:0006096)	8	55.95	4.45E-09
acetyl-CoA biosynthetic process from pyruvate (GO:0006086)	5	252.53	1.32E-07
lipid metabolic process (GO:0006629)	4	11.88	2.21E-01
biotin biosynthetic process (GO:0009102)	2	151.52	5.27E-01

*Adjusted *p*-value (Bonferroni correction).

Supplementary Figure 3. Co-expression network analysis identifies genes co-regulated with WRI1 in *Arabidopsis thaliana*. Co-expression measures were mined from a data set encompassing 18957 genes (ATTED-II database, see Methods). Starting from WRI1 as the first node (yellow), the network was extended by the five top-ranking WRI1 co-expressed genes (red nodes, red edges). These in turn were used as query genes and the network was further extended by the 50 top-ranking co-expressed genes in each case, adding 242 blue edges (white nodes). Of the added genes, 115 that are connected by only one node were removed to give a highly connected network module. **A**, resulting final network with 132 edges and 47 nodes (genes). **B**, showing only WRI1 with directly connected genes. **C**, GO term enrichment for gene set in (A). GO term analysis was performed using the DAVID version 2021 online resource (https://david.ncifcrf.gov/) (Huang da, W. et al. Nat Protoc 2009, 4: 44-57). Shown are the five top ranking terms for Biological Process. Network graphs were drawn with Graphviz (Ellson, J. et al., Graphviz and Dynagraph — Static and Dynamic Graph Drawing Tools. In M Jünger, P Mutzel, eds, Graph Drawing Software. Springer Berlin Heidelberg, Berlin, Heidelberg, pp 127-148, 2004). Gene names and IDs: ACAT2 (AT5G48230); ACLB-2 (AT5G49460); ACP1 (AT3G05020); ADK (AT5G50370); ATBIOF (AT5G04620); BADC1 (AT3G56130); BADC2 (AT1G52670); BADC3 (AT3G15690); BCA5 (AT4G33580); BCCP2 (AT5G15530); BIO2 (AT2G43360); CAC1 (AT5G16390); CAC2 (AT5G35360); CPI1 (AT5G50375); CTI1 (AT1G42960); DHLAT (AT1G34430); EMB3141 (AT5G50390); FAP3 (AT1G53520); FATA2 (AT4G13050); GPDHp (AT5G40610); HAD (AT5G10160); HAD2 (AT2G22230); KAR (AT1G24360); KASI (AT5G46290); KASIII (AT1G62640); KCS11 (AT2G26640); LPD1 (AT3G16950); LS (AT5G08415); LTA2 (AT3G25860); MCMT (AT2G30200); MOD1 (AT2G05990); MVA1 (AT4G11820); PDH-E1-ß(2) (AT2G34590); PDH-E1α (AT1G01090); PDH-E1β (AT1G30120); PGLM1 (AT1G22170); PKP1 (AT3G22960); PKP1 (AT5G52920); RUS6 (AT5G49820); SAD5 (AT3G02630); STKL1 (AT4G00238); TA (AT1G12230); WRI1 (AT3G54320).

		instrument output			final value
ligand	Sequence (forward strand)	k_d [nM]	Ec50 [nM]	response amplitude	K_d [nM]
● 242	GTTTAGT**C**T**T**TAGCTCTTA**CG**TTACGTT	72.9	no value	1.38	no binding
● 45	ATTTGTC**C**T**T**T**G**GTGGGAG**CG**AGCGGCC	331.8	19485	3.60	no binding
● 243	CTTTAGT**C**T**T**T**G**CGTCTTA**CG**AGTTACG	2999.8	no value	5.07	no binding
● 238	AAACCTA**C**C**T**C**G**AGACCAT**CG**TCTTCTT	52.6	57.97	16.60	52.6
● **16***	TGGGTTT**C**T**T**C**G**TAAGCAT**CG**AAATCAGA	16.0	20	16.99	16.0

*reference ligand used in each series of measurements

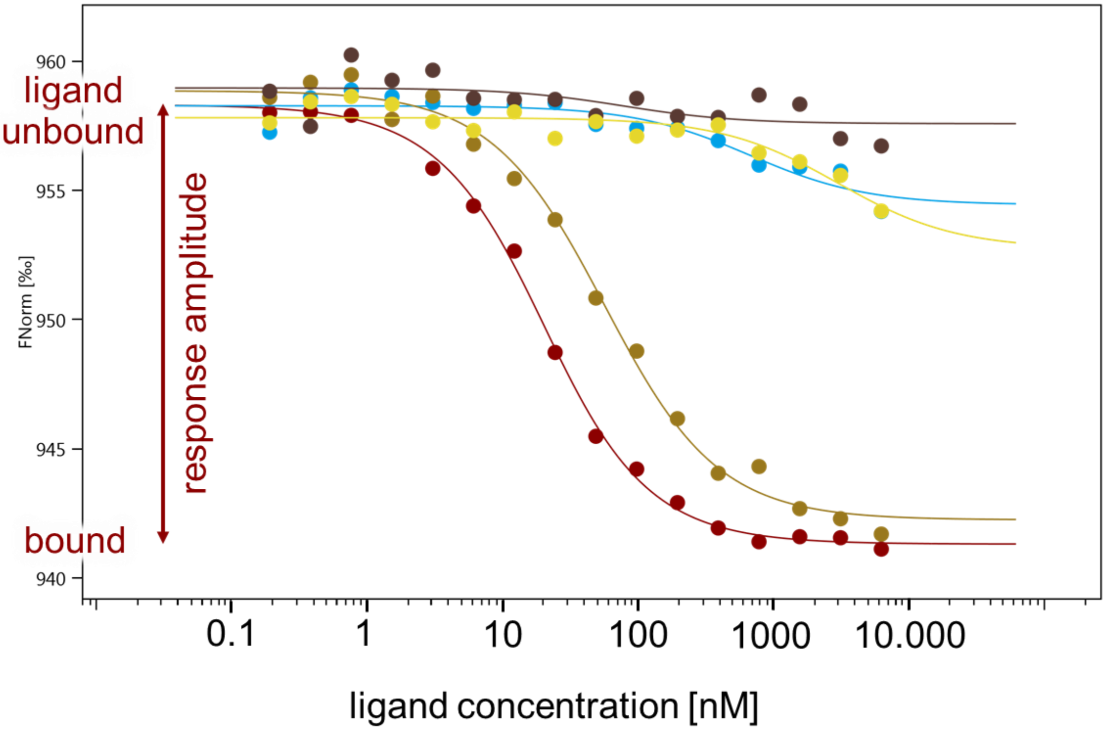

Supplementary Figure 4. Examples for binding curves of AtWRI1$_{58\text{-}240}$-GFP and 28 bp DNA ligands. Microscale Thermophoresis was performed with the Monolith NT.115 apparatus (NanoTemperTechnologies, South San Francisco, CA; nanotempertech.com). NanoTemper Analysis software v2.2.4 returned binding curves along with fitted k_d values, the concentration for which half of the ligand is bound (Ec50) as well as the response amplitude. If the response amplitude of a binding curve is not close to the response amplitude of the reference DNA probe (ligand 16), then the k_d value is rejected and the ligand is classified as non-binding. For acceptable determinations, K_d values and EC50 values also tend to be very similar.

A

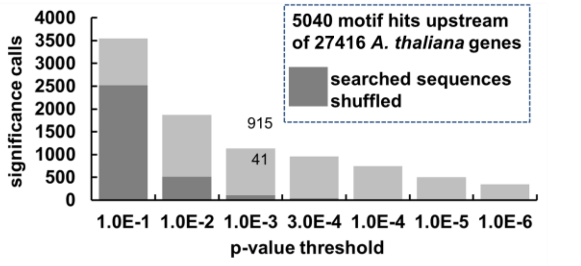

B

	motif / search pattern	p-value threshold	significance calls (5% FDR)
AW-box	C.T.G.......CG	3.0E-04	915
p1	G.T.C.......GC	4.0E-04	777
p2	G..C...CG....T	2.2E-04	707
p3	G...C..A..G..C	4.4E-04	803
p4	T..C...G..G..C	3.6E-04	692
p5	C...G....G..AC	2.3E-04	610
p6	G...C.G.....CT	4.5E-04	835
p7	CC....G.C....T	6.4E-04	1028
p8	T..GC....C...G	6.0E-05	443
p9	A....C.G..G..G	5.9E-04	1122
p10	G..G.....G.C.A	3.1E-04	882
		average:	789.9 ± 196.4

C

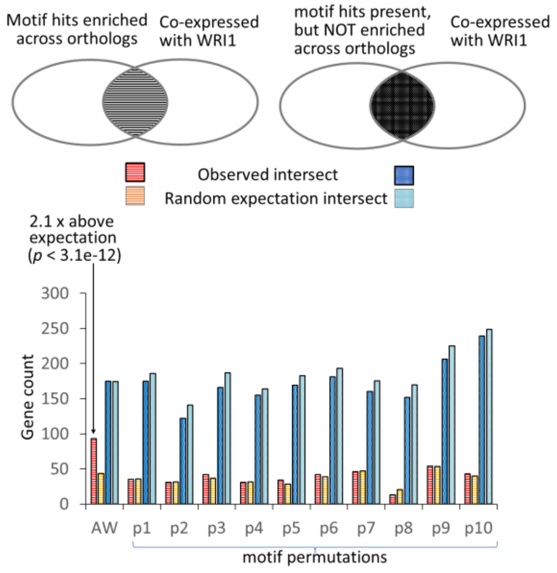

Supplementary Figure 5. Enrichment analysis of the AW-box consensus across orthologous upstream regions of 12 *Brassicaceae* genomes. A, Estimation of the False Discovery Rate (FDR) for sets of orthologous promoter regions (500 bp upstream ATG start) enriched with the AW-box by use of the empirical null model. The bars show the number of ortholog groups significantly enriched in dependence of the maximal hypergeometric *p*-value allowed (*p*-value threshold). The dark grey shading shows the number of significance calls if the motif detection was repeated after random shuffling of the searched sequences (3 repetitions were averaged). For *p*-values below $3.02x10^{-4}$ there are 915 significance calls with 41 null model calls (4.5%) (FDR < 5%). B, Number of significance calls if the same analysis was repeated with base substitutions and permutations of the AW-box. C, intersect between *A. thaliana* genes with AW-box hits and a set of 986 WRI1 co-expressed genes (see methods). Genes for which the motif is enriched across OURs have significant overlap with WRI1 co-expressed genes (hypergeometric *p*-value). For permutations of the AW-box motif (panel B), this enrichment is not observed. Genes with AW-box hits in the upstream region, but for which the motif is not enriched across orthologs, have no significant overlap.

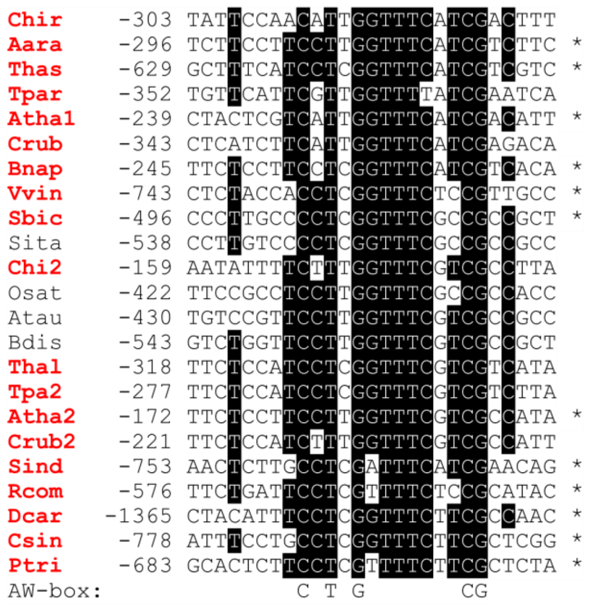

Supplementary Figure 6. Deeply conserved AW-box site in upstream regions of chloroplast isoforms for 2,3-bisphosphoglycerate-dependent phosphoglycerate mutase (PGLM) of mono- and eudicot plants. 23 aligned genomic sequences (19 plant species) were identified in the upstream regions of protein encoding genes annotated as 2,3-bisphosphoglycerate-dependent phosphoglycerate mutase. Names in red: The AW-box sequence information from the 12 *Brassicaceae* species (Supplementary Table 9) was extended by searches in upstream regions of non-*Brassicaceae* species for which syntenic orthology to the A. thaliana genes was verified (Supplementary Table 2). All shown sequences are listed in Supplementary Table 9 (AT1G22170, AT1G78050). Names in black: Additional PGLM genes of the monocots in *Setaria italica*, *Oryza sativa*, *Aegilops tauschii* and *Brachypodium distachyon* were analyzed as well, but synteny was not verified in these cases. Sequence position relative to the ATG start codon is shown. Residues identical for 70% or more of sequences in a given position are in white text on a black background. *, WRI1 binding affinity measured for this sequence (Supplementary Table 4, DNA fragments 35, 37, 139, 141-149; all k_d values < 50 nM). Abbreviations (species and protein sequence ID): Aara, AA_scaffold4989_34 (*Aethionema arabicum*, http://www.brassicadb.org); Atau, XP_020155880.1 (*Aegilops tauschii subsp. Tauschii*); Atha1, NP_564161.1 (*Arabidopsis thaliana*, PGLM1, AT1G22170); Atha2, NP_177928.2 (*Arabidopsis thaliana*, PGLM2, AT1G78050); Bdis, XP_003570632.1 (*Brachypodium distachyon*); Bnap, BnaA07g33900D (*Brassica napus*, http://www.genoscope.cns.fr/brassicanapus/); Chir, CARHR022520.1 (*Cardamine hirsuta*); Chir2, CARHR071500.1 (*Cardamine hirsuta*); Crub, XP_006305204.1 (*Capsella rubella*); Crub2, XP_006301178.1 (*Capsella rubella*); Csin, XP_006472637.1 (*Citrus sinensis*); Dcar, XP_017240349.1 (*Daucus carota subsp. sativus*); Osat, XP_015623414.1 (*Oryza sativa Japonica Group*); Ptri, XP_024450220.1 (*Populus trichocarpa*); Rcom, XP_002513886.1 (*Ricinus communis*); Sbic, XP_021315683.1 (*Sorghum bicolor*); Sind, XP_011074157.1 (*Sesamum indicum*); Sita, XP_004953943.1 (*Setaria italica*); Thal, XP_006390029.1 (*Eutrema salsugineum*); Thas, XP_010521196.1 (*Tarenaya hassleriana*); Tpar, Tp1g19670 (*Thellungiella parvula*, see ref. in Suppl. Table S1); Tpa2, Tp5g33180 (*Thellungiella parvula*, see ref. in Suppl. Table S1); Vvin, XP_019071742.1 (*Vitis vinifera*). Alignment was made using Clustal Omega online resource (Sievers, F. et al., Mol Syst Biol 2011, 7: 539) and BoxShade (https://embnet.vital-it.ch/software/BOX_form.html).

```
Atri    -137 TTATTTCCGATATTAACGAGGTTACCGA
Vvin    -397 TTATTTCCGAAGGCAACCAAGGTATTCA
Hann2   -145 GATTCTTCGATACACCCTAGGTACACCA
Hann1   -711 TTAAAATCGAGCCAAACCAAGCTGAGCC
Thas    -213 CGTCCTTCGATACGAACTAAGCTAAAGC
Grai    -218 ACTATTTCGATACAAACTAAGCACAAAG
Bnap    -123 TCTCTTTCGATACAAACTAAGCATTTAT
Alyr    -163 GTCGTTTCGATACGAACTAAGCAAATTC
Esal    -139 TCTCTTTCGATACGAACTAAGCAAATTC
Atha    -155 GCTTCTTCGATACGAACTAAGCACTTCT
Csin    -216 AAATCTTCGATACGAACTAAGCGCCTCT
Ptri    -180 TTTCCTTCGATACAAACAAAGCCAATCC
Rcom    -224 AATCCTTCGATACAAACTAAGCTTCTCC
Stub    -172 CCAGCTTCGATATTCACGAGGCAAGGCC
Pper    -179 GTTTATTCGATACAACCAAAGCCCTCCT
Dcar    -173 GCAGGTTCGATACAACCAAAGCCTCCCA
Mnot    -210 AATGGTTCGATATAACCAAAGCCTCCTC
Sbic    -183 AAGGCCTCGATACAAACAAGGCCGCCGC
Osat    -220 CCCCCTTCGATACAAACGAAGCCGCCGC
Hbra    -191 ATTTTTTCGATACAAACTAAGTCGAAGT
Tcac    -189 CTCTTGTCGATACAAACTAAGCTCTCTT
Jreg    -224 TCTTCTTCGATATAACCGAAGTTCCCTC
Jcur    -234 ATTTCTTCGATACAAACTAAGCTCCCTT
Mesc    -199 ATTTCTTCGATACAAACCAAGTTCCCTC
AW-box:             CG       C A G
```

Supplementary Figure 7. Deeply conserved AW-box site at 148 bp upstream of plastidic pyruvate kinase β_1-subunit (AT5G52920). The 24 aligned genomic sequences (23 plant species) were identified in the upstream regions of protein encoding genes annotated as "plastidial pyruvate kinase 2". Sequence position relative to the ATG start codon is shown. Residues identical for 70% or more of sequences in a given position are in white text on a black background. Abbreviations (species and protein sequence ID): Alyr, XP_002864200.1 (*Arabidopsis lyrata subsp. lyrata*); Atha, NP_200104.1 (*Arabidopsis thaliana*); Atri, XP_006843356.1 (*Amborella trichopoda*); Bnap, XP_022551725.1 (*Brassica napus*); Csin, XP_006490596.1 (*Citrus sinensis*); Dcar, XP_017259148.1 (*Daucus carota subsp. sativus*); Esal, XP_006401763.1 (*Eutrema salsugineum*); Grai, XP_012440062.1 (*Gossypium raimondii*); Hann1, XP_022000179.1 (*Helianthus annuus*); Hann2, XP_022006731.1 (*Helianthus annuus*); Hbra, XP_021655735.1 (*Hevea brasiliensis*); Jcur, XP_012090144.1 (*Jatropha curcas*); Jreg, XP_018847349.1 (*Juglans regia*); Mesc, XP_021592686.1 (*Manihot esculenta*); Mnot, XP_024022342.1 (*Morus notabilis*); Osat, XP_015622342.1 (*Oryza sativa Japonica Group*); Pper, XP_020413468.1 (*Prunus persica*); Ptri, XP_002317739.3 (*Populus trichocarpa*); Rcom, XP_002513613.1 (*Ricinus communis*); Sbic, XP_002458258.1 (*Sorghum bicolor*); Stub, XP_006366641.1 (*Solanum tuberosum*); Tcac, XP_007038892.2 (*Theobroma cacao*); Thas, XP_010555705.1 (*Tarenaya hassleriana*); Vvin, XP_010662621.1 (*Vitis vinifera*). Alignment was made using Clustal Omega online resource (Sievers, F. et al., Mol Syst Biol 2011, 7: 539) and BoxShade (https://embnet.vital-it.ch/software/BOX_form.html).

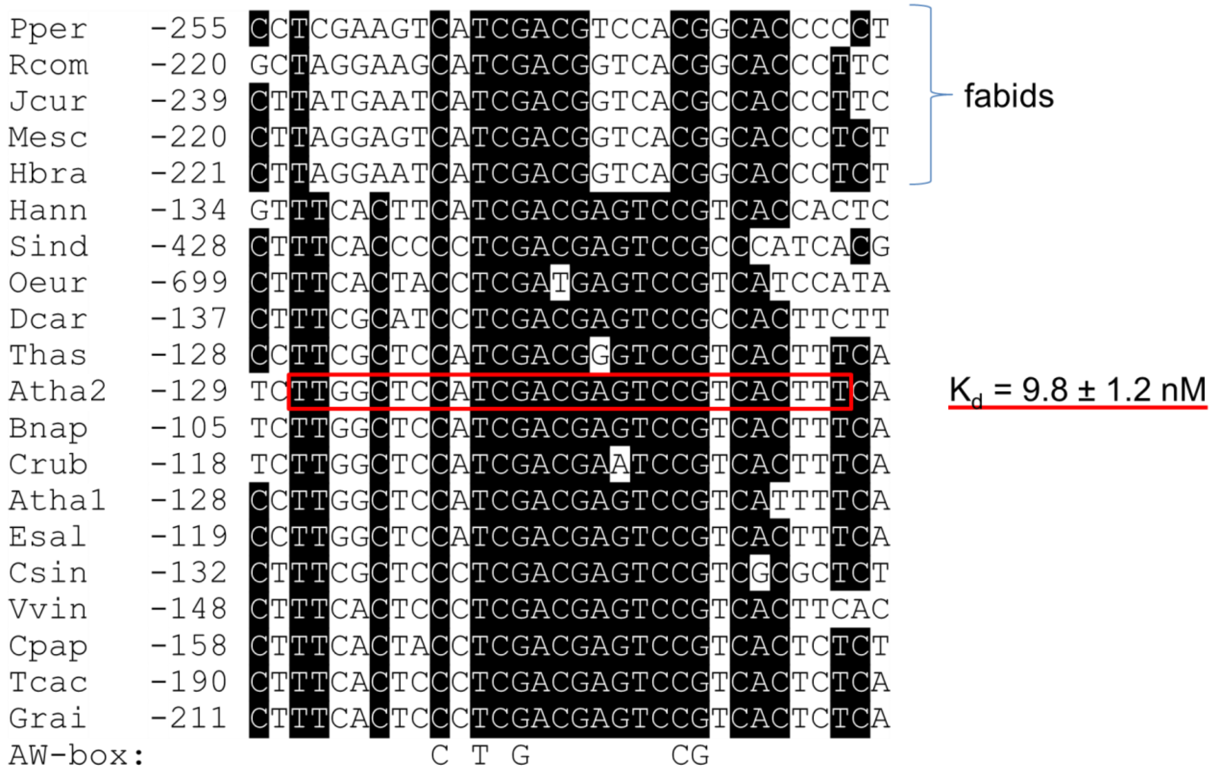

Supplementary Figure 8. Deeply conserved AW-box site in upstream regions of 6-phosphogluconate dehydrogenase (PGD) genes of eudicot plants. The 20 aligned genomic sequences (19 plant species) were identified in the upstream regions of protein encoding genes annotated as "6-phosphogluconate dehydrogenase, decarboxylating". Sequence position relative to the ATG start codon is shown. Residues identical for 70% or more of sequences in a given position are in white text on a black background. The red box marks an *A. thaliana* sequence for which a dissociation constant with WRI1 was measured by Microscale Thermophoresis (Supplementary Table 4, measurement 89). In support of the relatedness of the gene loci, the respective predicted protein sequences are highly similar: all pairwise alignments are un-gapped and amino acids identities are above 80 % (not shown). Protein sequences IDs: Atha1, NP_176601 (*Arabidopsis thaliana*, PGD1, AT1G64190); Atha2, NP_001318724 (*Arabidopsis thaliana*, PGD3, AT5G41670); Bnap, XP_013743462 (*Brassica napus*); Cpap, XP_021890969 (*Carica papaya*); Crub, XP_023634321 (*Capsella rubella*); Csin, XP_006476447 (*Citrus sinensis*); Dcar, XP_017223209 (*Daucus carota subsp. sativus*); Esal, XP_006391656 (*Eutrema salsugineum*); Grai, XP_012439737 (*Gossypium raimondii*); Hann, XP_022020208 (*Helianthus annuus*); Hbra, XP_021668644 (*Hevea brasiliensis*); Jcur, XP_012086783 (*Jatropha curcas*); Mesc, XP_021605174 (*Manihot esculenta*); Oeur, XP_022892974 (*Olea europaea var. sylvestris*); Pper, XP_020419402 (*Prunus persica*); Rcom, XP_002509902 (*Ricinus communis*); Sind, XP_011073556 (*Sesamum indicum*); Tcac, XP_017972824 (*Theobroma cacao*); Thas, XP_010545301 (*Tarenaya hassleriana*); Vvin, XP_002275970 (*Vitis vinifera*). Alignment was made using Clustal Omega online resource (Sievers, F. et al., Mol Syst Biol 2011, 7: 539) and BoxShade (https://embnet.vital-it.ch/software/BOX_form.html).
